# Pyruvate Kinase M2 Contributes to TLR-Mediated Inflammation and Autoimmunity by Promoting Pyk2 Activation

**DOI:** 10.3389/fimmu.2021.680068

**Published:** 2021-05-07

**Authors:** Xin Zhang, Yonghong Yang, Lina Jing, Weiwei Zhai, Hui Zhang, Qun Ma, Chunxia Li, Fenglian Yan, Dalei Cheng, Junfeng Zhang, Zhaochen Ning, Hui Shi, Changying Wang, Mingsheng Zhao, Jun Dai, Zhihua Li, Jiankuo Ming, Meimei Yu, Haiyan Wang, Hongyan Cheng, Huabao Xiong, Guanjun Dong

**Affiliations:** ^1^ School of Medical Laboratory, Weifang Medical University, Weifang, China; ^2^ Institute of Immunology and Molecular Medicine, Jining Medical University, Jining, China; ^3^ Medical Research Center, Affiliated Hospital of Jining Medical University, Jining, China; ^4^ Cheeloo College of Medicine, Shandong University, Jinan, China; ^5^ Department of Clinical Laboratory, Jining No. 1 People’s Hospital, Jining, China

**Keywords:** PKM2, TLR, Pyk2, inflammation, autoimmunity

## Abstract

Toll-like receptors (TLRs) play critical roles in regulating the abnormal activation of the immune cells resulting in the pathogenesis of inflammation and autoimmune diseases. Pyruvate kinase M2 (PKM2), which governs the last step of glycolysis, is involved in multiple cellular processes and pathological conditions. However, little is known about the involvement of PKM2 in regulating TLR-mediated inflammation and autoimmunity. Herein, we investigated the role of PKM2 in the activation of the TLR pathways and the pathogenesis of inflammation and autoimmune diseases. The activation of TLR4, TLR7 and TLR9 pathways was found to induce the up-regulation of PKM2 expression in macrophages, dendritic cells (DCs) and B cells. The over-expression of PKM2 promotes the activation of TLR4, TLR7 and TLR9 pathways while interference with the PKM2 expression or the addition of the PKM2 inhibitor (PKM-IN) markedly inhibited the activation of TLR4, TLR7 and TLR9 pathways. Mechanistically, PKM2 augmented the activation of TLR4, TLR7 and TLR9 pathways by promoting the activation of the proline-rich tyrosine kinase 2 (Pyk2). Intriguingly, the PKM2 inhibitor PKM2-IN significantly protected the mice from the endotoxic shock mediated by the TLR4-agonist LPS. Additionally, it alleviated the progression in the TLR7-agonist imiquimod-mediated lupus mice and spontaneous lupus MRL/*lpr* mice. Moreover, PKM2 expression was highly elevated in the monocytes, DCs and B cells from systemic lupus erythematous (SLE) patients compared with those from the healthy donors. Besides, the PKM2 expression level was positively correlated with the degree of activation of these immune cells. In summary, PKM2 contributed to TLR-mediated inflammation and autoimmunity and can be a valuable target to control inflammation and autoimmunity.

## Introduction

Toll-like receptors (TLRs) are an evolutionarily ancient family of pattern recognition receptors that regulate both innate and adaptive immune responses (1). Up to date, more than twenty TLRs have been identified in humans and animals; examples include TLR4, TLR7 and TLR9. As the “gatekeepers” of the immune system, TLRs play critical roles in protecting the host from invading bacteria, viruses and other microorganisms (2, 3). Furthermore, it has been demonstrated that the TLR-signaling pathway is crucial for the induction and progression of various diseases (4, 5). After binding to its ligand, TLRs recruit the myeloid differentiation factor 88 (MyD88) and other adaptor proteins to initiate innate immune cascades that participate in regulating the differentiation, activation and function of the immune cells (6). In particular, inappropriate signaling by TLR4, TLR7, and TLR9 induces hyper-activation of the immune system and contributes to numerous pathological conditions like inflammation and autoimmune diseases (7, 8). Therefore, tight regulation of the TLR-signaling pathways is crucial for preventing a hyperactive cellular phenotype that can result in inflammation and autoimmunity.

As is known, TLR4, TLR7 and TLR9 are commonly expressed on the macrophages, dendritic cells (DCs), and B lymphocytes (B cells). Numerous studies suggest that TLR4-, TLR7- and TLR9-mediated abnormal activation of the macrophages, DCs and B cells contributes to the pathogenesis of inflammation and autoimmune diseases (9–11). For example, the recognition of microbial pathogens and other inflammatory stimuli by TLRs on the DCs induces proinflammatory cytokines production and enhanced antigen presentation to naive T cells; subsequently, it activates antigen-specific adaptive immune responses (12, 13). TLR-activated monocytes/macrophages can infiltrate the target organ/s and contribute to systemic lupus erythematosus (SLE) pathogenesis through the production of proinflammatory cytokines (14). Moreover, TLR signaling plays a variety of roles in B cell development and activation. In addition to its function of promoting autoantibody production, TLR-stimulated B cell activation can contribute to SLE pathogenesis through antigen presentation as well as cytokine production (4, 15). However, the regulatory mechanism for activating the TLR pathway is extremely complex, and its specific molecular mechanism is still unclear. Thus, it is pivotal to explore the molecular mechanisms that participate in regulating the TLR signaling pathways to control the pathogenesis of inflammation and autoimmune diseases.

Pyruvate kinase M2 (PKM2), which governs the last step of glycolysis, is a rate-limiting enzyme possessing both metabolic and non-metabolic functions (16). PKM2 not only participates in tumor metabolic reprogramming as an important regulator but also plays an important role in the regulation of immune-inflammatory response mechanisms. Recent studies have revealed that PKM2 contributes to the activation of macrophages and the release of inflammatory factors (17, 18). Moreover, PKM2 detetramerization facilitates the metabolic rewiring for CD4^+^ T cell activation and T helper (Th) 1/Th17 cell development under *in vivo* and *in vitro* conditions (19, 20). Although it has been shown that PKM2 can regulate the activation of the TLR4 signaling pathway (21), there is no clarity regarding the role and mechanism of PKM2 in regulating the activation of TLR4, TLR7 and TLR9 signaling pathways in different immune cells and its involvement in the pathogenesis of inflammation and autoimmune diseases.

In the present study, we investigated the role of PKM2 in the TLR4-, TLR7- and TLR9-mediated activation of immune cells such as macrophages, DCs and B cells, and the pathogenesis of endotoxin shock and SLE. We found that TLR-induced up-regulation of PKM2 contributed to the activation of TLR4, TLR7 and TLR9 pathways by promoting the activation of proline-rich tyrosine kinase 2 (Pyk2). Intriguingly, pharmacological inhibition of PKM2 by PKM2-IN significantly protected the mice from the endotoxic shock mediated by the TLR4-agonist LPS. Moreover, it alleviated the progression in the TLR7-agonist imiquimod-mediated lupus mice and spontaneous lupus MRL/*lpr* mice. Besides, the PKM2 expression was abnormally elevated in patients with SLE, and it positively correlated with the degree of activation of the immune cells. Taken together, these results indicate that PKM2 contributes to the pathogenesis of inflammation and autoimmunity by promoting the activation of the TLR signaling pathways.

## Materials and Methods

### Animals

Wild type C57BL/6 mice in total between 6 weeks and 8 weeks of age were purchased from Pengyue Experimental Animal Breeding Company (China) and MRL/*lpr* mice (8-week old) were obtained from the Nanjing University Model Animal Research Centre. All the mice were maintained under specific pathogen-free conditions at Jining Medical University. For all experiments, mice were grouped according to genotype. Mice used to generate bone marrow-derived macrophages (BMDMs) and bone marrow-derived dendritic cells (BMDCs) were 6-10-week old. A topical dose of 1.25 mg of 5% imiquimod cream (Med-Shine Pharmaceutical, China) was applied 3 times weekly to the right ears of mice from the 8th week to 18th week. For systemic treatment, IMQ-induced lupus-prone mice received intraperitoneal injections, 3 times weekly, with 10 µg/g of PKM2-IN (Selleck) in 200 µl PBS from the 8th week to 18th week; MRL/*lpr* mice received 3 times weekly intraperitoneal injections with 10 µg/g of PKM2-IN from the 13th week to 18th week; 20-week old MRL/*lpr* mice received intraperitoneal injections with 10 µg/g of PKM2-IN every other day from two weeks. All experiments and procedures involving mice were performed in strict accordance with the Guiding Principles for the Care and Use of Laboratory Animals approved by the Jining Medica University Animal Care Committee.

### Antibodies

The following antibodies were used for immunoblotting: Cell Signaling Technology, anti-p38 (Cat#: 8690), anti-p-p38 (Cat#: 4511), anti-p65 (Cat#: 8242), anti-p-p65 (Cat#: 3033), anti-JNK (Cat#: 9252), anti-p-JNK (Cat#: 4668), anti-Erk (Cat#: 4695), anti-p-Erk (Cat#: 4370), anti-PKM2 (Cat#: 4053), anti-STAT3 (Cat#: 12640); Abcam, anti-Pyk2 (Cat#: 228477); Santa Cruz Biotechnology, anti-p-Pyk2 (Cat#: sc-81512); Beyotime Institute of Biotechnology, anti-β-actin (Cat#: AA128), HRP labeled Goat Anti-Rabbit IgG (Cat#: A0208), HRP-labeled Goat Anti-Mouse IgG (Cat#: A0216). The following antibodies purchased from Biolegend were used for flow cytometry: FITC anti-mouse B220 (Cat#: 103206), BV421 anti-mouse CD11c (Cat#: 117330), FITC anti-mouse F4/80 (Cat#: 123108), APC anti-mouse CD86 (Cat#: 105012), PE anti-mouse CD40 (Cat#: 124610), PE anti-mouse GL7 (Cat#: 144607), APC anti-mouse CD95 (Cat#: 152604), APC anti-mouse CXCR5 (Cat#: 145506), PE anti-mouse PD-1 (Cat#: 135206), APC/Cy7 anti-human CD19 (Cat#: 302218), FITC anti-human CD14 (Cat#: 367116), BV421 anti-human CD11c (Cat#: 301628), APC anti-human CD86 (Cat#: 374208), PE anti-human CD40 (Cat#: 334308). All the antibodies for flow cytometry were used at a 1:100 dilution.

### Preparation of Bone Marrow-Derived Macrophages (BMDMs) and Dendritic Cells (BMDCs)

BMDMs and BMDCs were obtained as previously described (22). Briefly, the bone marrow cells was fleshed from tibias and femurs of C57BL/6 mice and cultured in complete Dulbecco’s Modified Eagle Medium (DMEM, Gibco) with granulocyte-macrophage colony stimulating factor (10 ng/mL) (GM-CSF, Peprotech) for BMDM differentiation, or in complete RPMI 1640 medium with GM-CSF (20 ng/mL) and IL-4 (1 ng/mL). For stimulation, LPS (100 ng/mL, Sigma), R848 (1 µg/mL, MCE), CpG-1826 (1 µM, Invitrogen), PKM2-IN (2.5, 5 and 10 µM, Selleck) and TAE-226 (1, 2, 4 µM, Selleck) were used.

### Isolations of Murine Splenic B Cells

Primary splenic murine B cells were isolated *via* negative selection using a mouse B cell isolation kit (Miltenyi Biotec) and the purity of B cells was always above 95%. The sorted B cells were cultured in RPMI 1640 medium containing 10% fetal bovine serum for the subsequent study.

### Cell Viability Assay

Cell viability was determined by CCK-8 assay (Beyotime). BMDMs and BMDCs were seeded in 96-well plate and incubated with different concentrations of PKM2-IN (5 μM, 10 μM, 20 μM, 40 μM, 80 μM and 160 μM) for 24 hours. Murine splenic B cells were seeded in 96-well plate and incubated with different concentrations of PKM2-IN (0.5 μM, 1 μM, 2 μM, 4 μM, 8 μM and 16 μM) for 24 hours. Cell viability was then measured according to the manufacturer’s protocol. Absorbance was measured at 450 nm in a microplate reader (BioTek).

### Murine Model of Endotoxic Shock

Female C57BL/6 mice in total between 8 weeks and 10 weeks of age were randomly distributed into different groups. The mice were intraperitoneally injected with various doses of PKM2-IN (5, 10 and 20 µg/g body weight) for 2 hours before LPS (10 μg/g body weight) injection. Serum samples were collected after 3 hours, flow cytometry and histological analyses(livers, lungs, and spleens)were collected after 12 hours. To determine the survival rate, mortality were monitored every hour for 120 hours after injecting with LPS (37.5 µg/g body weight), after which no further loss of mice occurred.

### Quantitative Real-Time PCR Analysis

Total RNA was isolated from cells using TRIzol reagent (Invitrogen). Complementary DNAs (cDNAs) were synthesized from total RNA using a RevertAid First Strand cDNA Synthesis Kit (ThermoFisher Scientific). DNA was synthesized from cDNA using a SYBR Green PCR Master Mix (Vazyme Biotech). All the expression of mRNA was normalized to GAPDH mRNA expression and the fold-changes were quantified using the 2^−ΔΔCt^ method.

### H&E Staining

Excised lung, liver and kidney tissues were fixed in 4% paraformaldehyde for 24 hours. Dehydrated with increasing concentrations of ethanol, embedded in paraffin then cut into 4 μm sections. The slides were stained with hematoxylin and eosin and were observed under an optical microscope (Nikon Corporation, Tokyo, Japan) in randomly selected fields.

### Immunofluorescence Staining

Briefly, a series of xylene and ethanol dewaxed and rehydrated tissue sections were used. For IgG and IgM detections, the kidney tissue sections were blocked with 1% bovine serum albumin (BSA) and incubated with Alexa Fluor 488-conjugated goat antimouse IgG or Alexa Fluor 488-conjugated goat anti-mouse IgM overnight at 4°C. The next day, after washed by PBS with 0.1% Tween 20, the sections were stained with DAPI and then sealed the cover slips with anti-fluorescence quenching agent. For PKM2 detections, after blocking with 1% BSA, the tissue sections were incubated with anti-PKM2 primary antibodies overnight at 4°C. The next day, after washed by PBS with 0.1% Tween 20, the sections were stained with Alexa Fluor 488-conjugated goat anti-mouse IgG for 1 hour at room temperature and then sealed the cover slips with anti-fluorescence quenching agent. Finally, the sections were analyzed under a fluorescence microscope (Olympus, Japan).

### Lentivirus Infections

Lentiviruses expressing PKM2 (PKM2-LV) and lentiviruses expressing PKM2-specific RNAi (RNAi-PKM2-LV) were purchased from GeneChem (China) and used to infect BMDMs following the standard protocols. Briefly, on day 3 during the induction of BMDMs, the cells were infected with 100 multiplicity of infection (MOI) PKM2-LV, RNAi-PKM2-LV or negative control-LV. After 24 hours, stale medium were replaced by fresh medium with murine GM-CSF (10 ng/mL). On day 7, BMDMs were collected and used for subsequent experiments.

### Isolation of Human Peripheral Blood Mononuclear Cells (PBMCs)

Blood from SLE patients and healthy subjects was collected in heparinized tubes, and PBMCs were obtained using Ficoll-Paque™ PLUS (GE Healthcare). All patients met the American College of Rheumatology 1997 update to the 1982 criteria for the classification of lupus and the study protocol was approved by the ethics committee at Jining Medical University. Under certain circumstances, for generation of monocyte-derived macrophages (MDMs), PBMCs isolated from healthy donors were cultured in RPMI 1640 medium containing 10% FBS and human GM-CSF (10 ng/mL) for 7 days. Human CD19^+^ B cells were isolated from healthy donors PBMCs and cultured in RPMI 1640 medium containing 10% FBS. For stimulation, LPS (100 ng/mL), R848 (1 µg/mL), CpG-2006S (1 µM), PKM2-IN (10 µM) were used.

### *In Situ* Terminal Deoxynucleotidyl Transferase-Mediated Uridine Triphosphate Nick-End Labeling Assay

TUNEL staining was performed with the manufacturer’s protocol (Roche, Switzerland). The protocol was followed exactly.

### Flow Cytometry

For surface staining, single-cell suspensions were stained with antibodies for 30 min at 4°C and then washed twice with 2 mL of PBS. The collected cells were resuspended in 0.3 mL of PBS and data were acquired with a FACS Canto II (Becton Dickinson, USA).

For intracellular staining, after surface-stained with antibodies and washed with PBS, cells were resuspended in 800 µl IC Fixation Buffer (eBioscience, USA) and incubated for 30 min at 4°C in the dark. After washed twice with 2 mL Permeabilization Buffer (eBioscience, USA), intracellular staining was performed in 100 µl of Permeabilization Buffer by using anti-mouse PKM2 antibody. After incubating overnight in the dark at 4°C, cells were washed for twice with 2 mL Permeabilization Buffer. Flow cytometric acquisition was performed on FACS Canto II (Becton Dickinson, USA). Analysis was performed using FlowJo software. An isotype control was used for each antibody.

### Enzyme-Linked Immunosorbent Assay

The IL-6 and TNF-α concentration was measured using mouse IL-6 or TNF-α ELISA kits (Biolegend), and the IL-12 concentration was measured using the mouse IL-12 ELISA kit (Dakewe Biotech, China) according to the manufacturer’s instructions. Optical density (OD) values at 450 nm were read using an ELx800 Absorbance Microplate Reader (BioTek, VT, USA). Two replicate wells were quantified for every sample, and all experiments were performed in triplicate.

### Co-Immunoprecipitation

The BMDMs were stimulated with LPS for 1 or 2 hours and then lysed in a modified RIPA buffer containing 1 mM PMSF and 1× protease inhibitor cocktail. The Co-IP assay was then performed as previously described. Briefly, the supernatant was separately incubated with anti-PKM2 antibody, anti-Pyk2 antibody or negative control IgG antibody followed by pull-down with 30 µl Protein A Agarose beads (cell signaling technology, USA). The beads were then collected by centrifugation at 12,000 rpm for 2 min and washed three times with cold PBS. The immunoprecipitates were eluted by boiling in 1× loading buffer for 10 min and subjected to western blot analysis along with input sample as described above.

### Immunoblotting Analysis

Protein was subjected to SDS-polyacrylamide gel electrophoresis and was transferred onto 0.45 µm PVDF membranes (Millipore). Membranes were then incubated with primary antibodies overnight at 4°C. Horseradish peroxidase (HRP)-conjugated secondary antibodies were used as the secondary detection antibodies. Finally, ECL plus western blotting detection reagents (ThermoFisher Scientific) were used to visualize protein expression. All the expression levels of protein were normalized to the β-actin level. Band intensities on the western blots were quantified by using ImageJ software.

### Adoptive Transfer and Endotoxic Shock

Female C57BL/6 mice were intraperitoneally injected with CFSE-labeled vehicle-treated BMDMs or PKM2-IN-treated BMDMs (2×10^6^ cells/mouse) for 12 hours. To determine the survival rate, these mice were injected with LPS (37.5 µg/g body weight) and the mortality was monitored every hour for 120 hours, after which no further loss of mice occurred. To analyze histological damage, these mice were injected with LPS (10 µg/g body weight), and the livers, lungs and spleens were collected after 12 hours.

### Statistical Analysis

Statistical tests used are indicated in figure legends. All data are analyzed using Prism software (GraphPad) and shown as means ± S.E.M. Differences between data sets were analyzed by performing ANOVA test or *t*-test. Differences were considered significant if *p*≤ 0.05.

## Results

### Activation of the TLR4/TLR7/TLR9 Pathways Induces the Up-Regulation of PKM2 Expression

**Figure 1:**
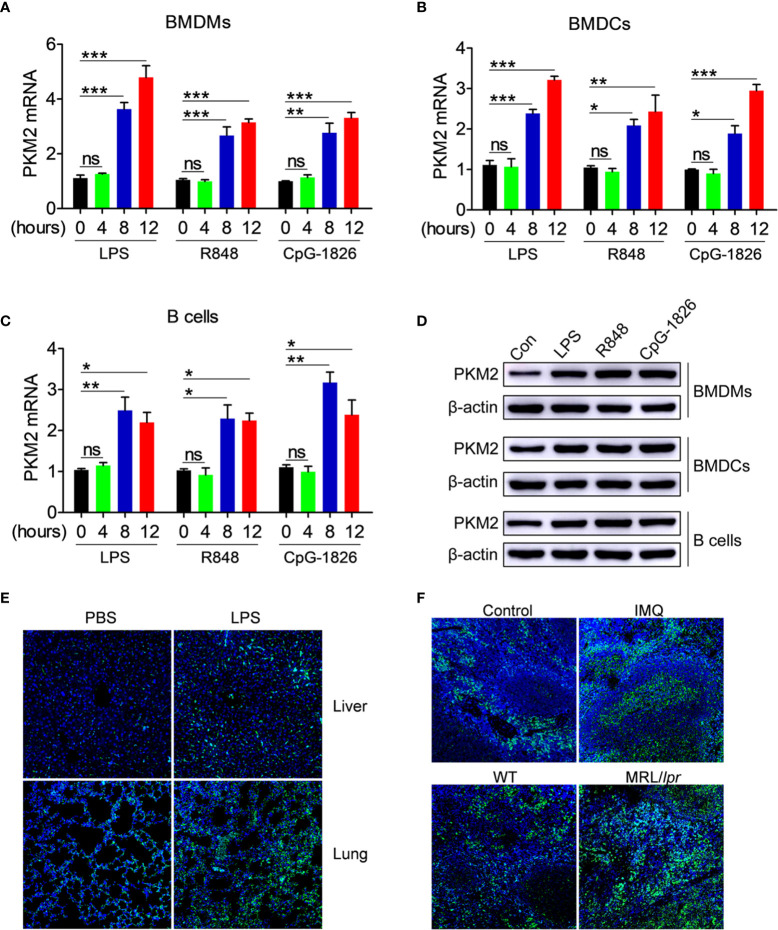
PKM2 expression is induced by TLRs *in vitro* and *in vivo*. **(A–D)** BMDMs, BMDCs and murine splenic B cells were stimulated with LPS (100 ng/mL), R848 (1μg/mL) and CpG-1826 (1 μM) for different times. Q-PCR analysis of PKM2 expression in BMDMs **(A)** BMDCs **(B)** and murine splenic B cells **(C)** at 4, 8 and 12 hours. Western blot analysis of PKM2 expression in BMDMs, BMDCs and murine splenic B cells at 24 hours **(D)**. The data shown represent the means of three independent experiments and the error bars represent the S.E.M. **(E)** Immunofluorescence staining to detect the expression of PKM2 in the liver and lung sections from mice challenged by LPS (10 μg/g body weight) for 12 hours. **(F)** Immunofluorescence staining to detect the expression of PKM2 in the spleen sections from mice treated by imiquimod for 8 weeks or lupus-prone MRL/*lpr* mice. Blue represents DAPI; green represents PKM2. The data are shown as the means ± SEM (n = 6 mice/group) and are representative of three independent experiments. *p < 0.05, **p < 0.01, ***p < 0.001, as determined by ANOVA tests; ns denotes *p* > 0.05.

The role of abnormal activation of TLR4, TLR7 and TLR9 pathways in regulating the PKM2 expression was explored initially. As shown in **Figures 1A–C**, the TLR4-agonist LPS, TLR7-agonist R848 and TLR9-agonist CpG–1826 significantly promoted the PKM2 mRNA expression in a time-dependent manner in the BMDMs (**Figure 1A**), BMDCs (**Figure 1B**) and B cells (**Figure 1C**). The same phenomenon was also observed at the protein level by the western blot (**Figures 1D** and **S1**). To explore the role of activation of TLR pathways in promoting PKM2 expression under *in vivo* conditions, the level of PKM2 in the LPS- or TLR7-agonist imiquimod-treated mice was examined. As shown in **Figure 1E**, compared with the non-treated mice, the mice challenged with LPS showed higher levels of PKM2 expression in the liver and lung tissues. Likewise, the expression of PKM2 in the spleen from imiquimod-treated mice was markedly increased compared with that in the control mice (**Figure 1F**). Moreover, the PKM2 expression was also abnormally elevated in the spleens of the spontaneous lupus MRL/*lpr* mice compared with that in the control mice (**Figure 1F**). The above studies suggested that the activation of the TLR pathways induced the PKM2 expression under *in vivo* and *in vitro* conditions.

### PKM2 Augments the Activation of TLR4/TLR7/TLR9 Pathways in Macrophages Under *In Vitro* Conditions

**Figure 2:**
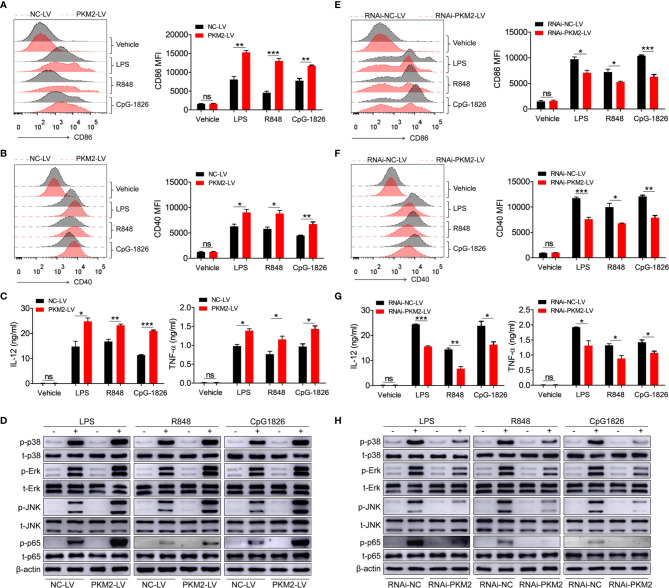
PKM2 augments TLRs-induced activation of BMDMs. **(A–D)** BMDMs, infected with lentivirus expressing PKM2 (PKM2-LV) or negative control lentivirus (NC-LV) for 3 days, were stimulated by LPS (100 ng/mL), R848 (1μg/mL) and CpG 1826 (1 μM). FACS analysis of CD86 **(A)** and CD40 **(B)** expression at 24 hours. ELISA analysis of the levels of IL-12 and TNF-α secreted by BMDMs at 24 hours **(C)**. Western blot analysis of the phosphorylation levels of p38, Erk, JNK, and p65 at 1 hour **(D)**. **(E–H)** BMDMs, infected with lentivirus expressing negative control-RNAi (RNAi-NC-LV) or PKM2-specific RNAi (RNAi-PKM2-LV) for 3 days, were stimulated by LPS (100 ng/mL), R848 (1μg/mL) and CpG 1826 (1 μM). FACS analysis of CD86 **(E)** and CD40 **(F)** expression at 24 hours. ELISA analysis of the levels of IL-12 and TNF-α secreted by BMDMs at 24 hours **(G)**. Western blot analysis of the phosphorylation levels of p38, Erk, JNK, and p65 at 1 hour **(H)**. The data shown represent the means of three independent experiments and the error bars represent the S.E.M. *p < 0.05, **p < 0.01, ***p < 0.001, as determined by ANOVA tests; ns denotes *p* > 0.05.

To explore the role of PKM2 in regulating the activation of the TLR4/TLR7/TLR9 pathways, PKM2-OE lentiviruses (PKM2-LV) were constructed for the over-expression of PKM2. As shown in **Figures S2A, B**, BMDMs infected with PKM2-LV showed significantly higher level of PKM2 compared with that infected with NC-LV. Intriguingly, the over-expression of PKM2 in the BMDMs by transfection with PKM2-LV significantly promoted the LPS-, R848- and CpG-1826-induced expressions of CD86 (**Figure 2A**) and CD40 (**Figure 2B**). Moreover, the levels of IL-12 and TNF-α secreted under the influence of LPS, R848 or CpG–1826 (**Figure 2C**) were higher in the BMDMs transfected with the PKM2-LV compared with the levels in the BMDMs transfected with NC-LV. In addition, the effect of PKM2 on the TLR4/TLR7/TLR9-induced activation of MAPKs and NF-κB pathways was also analyzed. Consistently, the over-expression of PKM2 significantly promoted the LPS-, R848- or CpG–1826-induced phosphorylations of p38, Erk, JNK, and p65 (**Figure 2D** and **Figures S3A–D**).

To further confirm the regulatory function of PKM2 on the activation of the TLR4/TLR7/TLR9 pathways, RNAi-PKM2 lentiviruses were constructed to knock down the expression of PKM2. As shown in **Figures S2C, D**, BMDMs infected with RNAi-PKM2-LV showed significantly lower level of PKM2 compared with that infected with RNAi-NC-LV. As expected, PKM2 knockdown in the BMDMs markedly reduced the LPS-, R848- and CpG–1826-induced expressions of CD86 (**Figure 2E**) and CD40 (**Figure 2F**), as well as the secretions of IL-12 and TNF-α (**Figure 2G**). Importantly, PKM2 knockdown significantly inhibited the LPS-, R848- or CpG–1826-induced phosphorylations of p38, Erk, JNK, and p65 (**Figures 2H** and **S3E–H**). All these data demonstrated that PKM2 augmented the activation of the TLR4/TLR7/TLR9 pathways.

### Pharmacological Inhibition of PKM2 Reduces the Activation of the TLR4/TLR7/TLR9 Pathways in BMDMs, BMDCs, and B Cells

**Figure 3:**
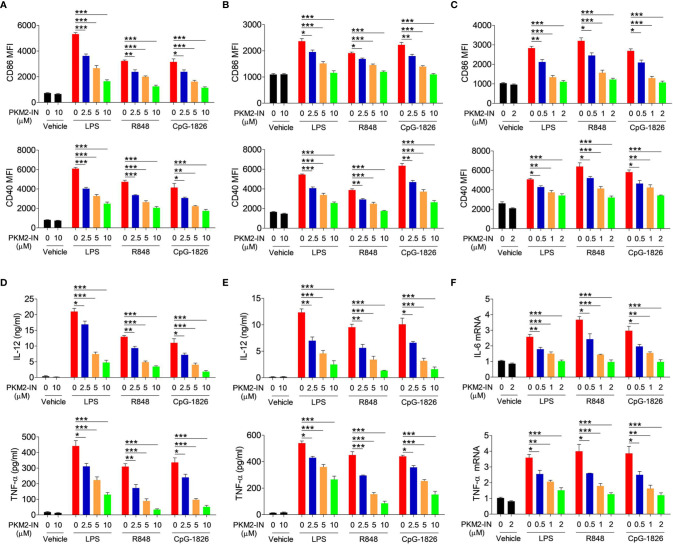
Pharmaceutical inhibition of PKM2 inhibits TLRs-induced activations of BMDMs, BMDCs and murine splenic B cells. BMDMs, BMDCs and murine splenic B cells were pretreated with PKM2 inhibitor PKM2-IN (2.5, 5 and 10 μM) for 2 hours followed by stimulations of LPS (100 ng/mL), R848 (1 μg/mL) and CpG-1826 (1 μM). FACS analysis of CD86 and CD40 expressions on BMDMs **(A)**, BMDCs **(B)** and murine splenic B cells **(C)** at 24 hours. ELISA analysis of the levels of IL-12 and TNF-α secreted by BMDMs **(D)** and BMDCs **(E)** at 24 hours. Q-PCR analysis of the mRNA levels of IL-6 and TNF-α on murine splenic B cells at 6 hours. The data shown represent the means of three independent experiments and the error bars represent the S.E.M. *p < 0.05, **p < 0.01, ***p < 0.001, as determined by ANOVA tests.

The PKM2 inhibitor PKM2-IN was employed to evaluate the effect of PKM2 on regulating the TLR4/TLR7/TLR9-induced activation of BMDMs, BMDCs, and B cells, respectively. Importantly, PKM2-IN did not affect the viability of BMDMs and BMDCs when the concentration was below 80 µM (**Figures S4A, B**) and the viability of B cells when the concentration was below 16 µM (**Figure S4C**). As shown in **Figure 3A**, the PKM2-IN pretreatment significantly reduced the LPS-, R848- and CpG-1826-induced expressions of CD86 and CD40 in a dose-dependent manner in BMDMs. Consistently, similar phenomena were also found in BMDCs (**Figure 3B**) and B cells (**Figure 3C**).

The effect of the PKM2-IN on the TLR4/TLR7/TLR9-induced expressions of the proinflammatory cytokines was also analyzed in BMDMs, BMDCs and B cells. As expected, PKM2-IN significantly inhibited the LPS-, R848- and CpG-1826-induced secretions of IL-12 and TNF-α by BMDMs and BMDCs in a concentration-dependent manner (**Figures 3D, E**). Moreover, PKM2-IN also significantly inhibited the LPS-, R848- and CpG-1826-induced mRNA levels of IL-12 and TNF-α by BMDMs and BMDCs in a concentration-dependent manner (**Figure S5**). The same phenomenon was also observed in B cells by Q-PCR (**Figure 3F**). From these experiments, it was concluded that blocking of PKM2 significantly inhibited the TLR4/TLR7/TLR9-induced activation of the BMDMs, BMDCs and B cells.

### Pharmacological Inhibition of PKM2 Inhibits the TLR4/TLR7/TLR9-Mediated Activation of the MAPK and NF-κB Pathways

**Figure 4:**
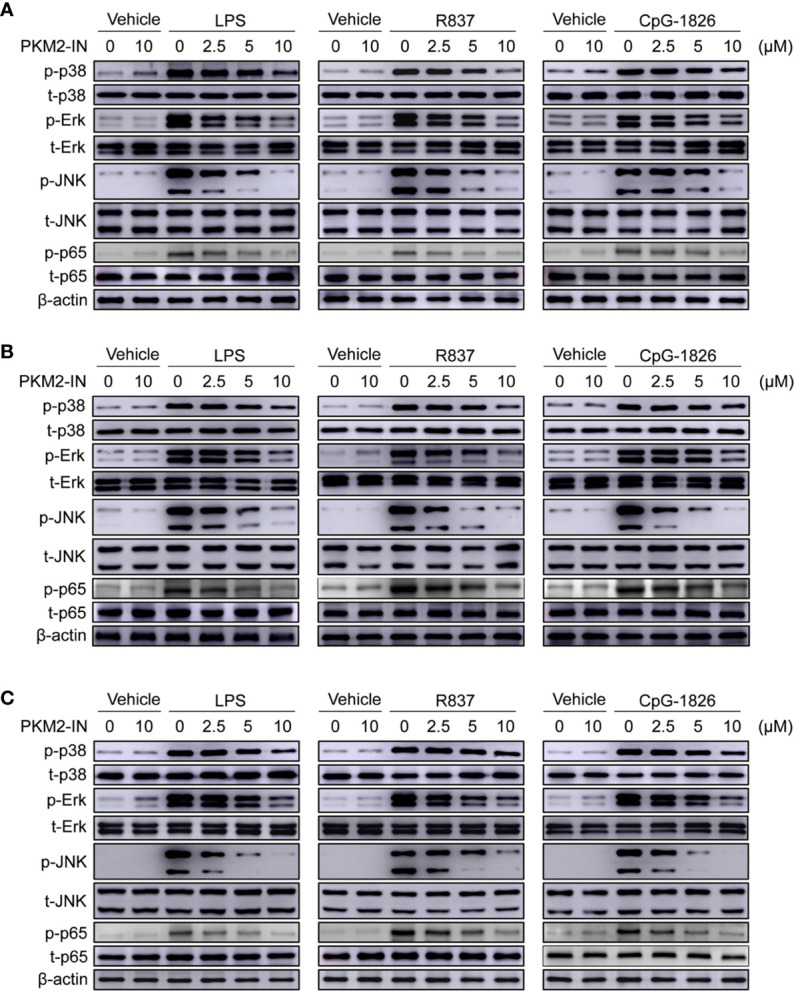
Pharmaceutical inhibition of PKM2 inhibits TLRs-induced activations of MAPKs and NF-κB pathways in BMDMs, BMDCs and murine splenic B cells. BMDMs, BMDCs and murine splenic B cells were pretreated with PKM2 inhibitor PKM2-IN (2.5, 5 and 10 μM) for 2 hours followed by stimulations of LPS (100 ng/mL), R848 (1 μg/mL) and CpG-1826 (1 μM). Western blot analysis of the phosphorylation levels of p38, Erk, JNK, and p65 in BMDMs **(A)**, BMDCs **(B)** and murine splenic B cells **(C)** at 1 hour. Data shown are representative of three independent experiments.

Next, the role of PKM2-IN in the activation of MAPK and NF-κB pathways was investigated in the presence of TLR4/TLR7/TLR9 in BMDMs, BMDCs and B cells. As shown in **Figure 4A** and **Figures S6A–D**, PKM2-IN significantly inhibited the LPS-, R848- and CpG–1826-induced phosphorylations of p38, Erk, JNK, and p65 in BMDMs in a concentration-dependent manner. Moreover, PKM2-IN also significantly inhibited the phosphorylations of p38, Erk, JNK, and p65 in BMDCs and B cells in a concentration-dependent manner (**Figures 4B, C** and **S6E–L**). Taken together, these data demonstrated that PKM2 alleviated the activation of TLR4/TLR7/TLR9 pathways in BMDMs, BMDCs and B cells.

### Pyk2 Is Involved in the Promotion Function of PKM2 on the Activation of the TLR4/TLR7/TLR9 Pathways

**Figure 5:**
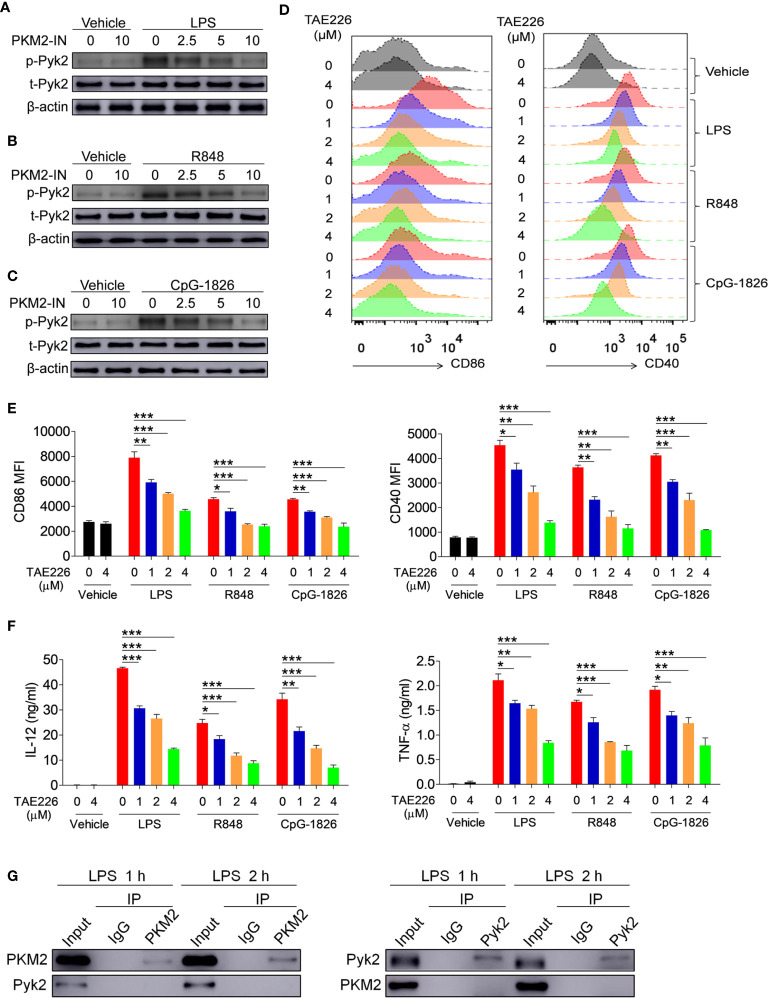
PKM2 augments the activations of TLR4/TLR7/TLR9 pathways by promoting Pyk2 activation. **(A–C)** BMDMs were treated with PKM2-IN (2.5, 5, 10 μM) for 2 hours followed by stimulations of LPS (100 ng/mL), R848 (1 μg/mL) and CpG-1826 (1 μM). Western blot analysis of the phosphorylation levels of Pyk2 at 1 hour. **(D–F)** BMDMs were treated with TAE226 (1, 2, 4 μM) for 2 hours followed by stimulations of LPS (100 ng/mL), R848 (1 μg/mL) and CpG-1826 (1 μM). FACS analysis of CD86 and CD40 expression at 24 hours **(D, E)**. ELISA analysis of the levels of IL-12 and TNF-α secreted by BMDMs at 24 hours **(F)**. **(G)** Co-IP analysis of the interaction between PKM2 and Pyk2. The data shown represent the means of three independent experiments and the error bars represent the S.E.M. *p < 0.05, **p < 0.01, ***p < 0.001, as determined by ANOVA tests.

Considering that Pyk2 is involved in the regulation of LPS-induced downstream pathway activation (23, 24), it was hypothesized that PKM2 may promote the activation of the TLR4/TLR7/TLR9 pathways by promoting Pyk2 activation. To test this, the effect of PKM2 was analyzed on the TLR-induced phosphorylation of Pyk2. As shown in **Figures 5A–C**, activation of the TLR4/TLR7/TLR9 pathways induced the phosphorylation of Pyk2, whereas the addition of PKM2-IN reversed the TLR4/TLR7/TLR9-induced Pyk2 phosphorylation in BMDMs, BMDCs and B cells. Thus, PKM2 was involved in regulating the Pyk2 activity. Furthermore, TAE226, a Pyk2 inhibitor, significantly inhibited TLR4/TLR7/TLR9-induced expressions of CD86 and CD40 on the BMDMs (**Figures 5D, E**). Additionally, it reduced the secretions of IL-12 and TNF-α induced by the TLR4/TLR7/TLR9 pathways (**Figure 5F**), as well as the mRNA levels of IL-12 and TNF-α (**Figure S7**). Next, co-immunoprecipitation (Co-IP) experiments were carried out to investigate the possibility of PKM2 binding to PYK2 and affect its activity. The results demonstrated that PKM2 did not bind with Pyk2 (**Figure 5G**). Of note, it has been shown that PKM2 can interact with STAT3 and ERK1/2 to play its function. As shown in **Figure S8**, although no evidence showed that PKM2 could interact with STAT3, PKM2 could interact with ERK, which participates in the activation of TLRs signaling pathways, indicating that the interaction between PKM2 and ERK maybe involved in the promotion function of PKM2 on the activation of the TLR pathways. All these data suggested that Pyk2 and ERK are involved in the promotion function of PKM2 on the activation of the TLR4/TLR7/TLR9 pathways.

### Pharmacological Inhibition of PKM2 Alleviated the LPS-Induced Endotoxin Shock

**Figure 6:**
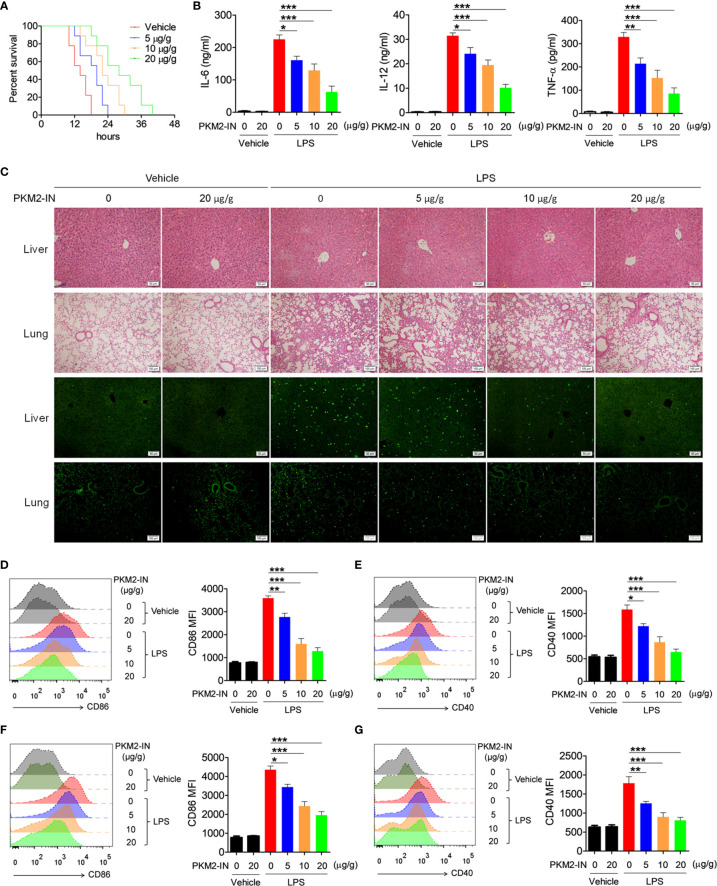
Pharmaceutical inhibition of PKM2 relieves LPS-induced endotoxin shock. **(A)** C57BL/6 mice were intraperitoneally injected with PKM2-IN (5, 10, 20 μg/g body weight) or vehicle for 2 hours followed by LPS challenge (37.5 μg/g body weight) and the mortalities of mice were observed (n=9 mice/group). **(B–G)** C57BL/6 mice were intraperitoneally injected with PKM2-IN (5, 10, 20 μg/g body weight) or vehicle for 2 hours followed by LPS challenge (10 μg/g body weight). Levels of IL-6, IL-12 and TNF-α in serum were determined by ELISA after 3 hours **(B).** Lung and liver sections were stained with H&E and apoptosis in the cells of lung and liver was analyzed by TUNEL **(C)**. FACS analysis was performed to assess the expressions of CD86 and CD40 on surface of splenic F4/80^+^ macrophages **(D, E)** and CD11c^+^ DCs **(F, G)** after 12 hours. The data are shown as the means ± SEM (n=6 mice/group) and are representative of three independent experiments. *p < 0.05, **p < 0.01, ***p < 0.001, as determined by ANOVA tests.

Since PKM2 positively regulates the activation of the TLR4/TLR7/TLR9 pathways under *in vitro* conditions, the effect of PKM2 in relieving the TLR-mediated inflammation and autoimmune diseases was assessed. Firstly, the effects of PKM2-IN on LPS-induced endotoxin shock were investigated. As shown in **Figure 6A**, intraperitoneal injection of PKM2-IN significantly reduced the mortality of the mice challenged by LPS; the higher the dose of PKM2-IN injection, the better was the survival of the mice within a certain dose range. Furthermore, endotoxin-shock-mice administered with PKM2-IN showed significantly reduced serum levels of IL-12 and TNF-α compared with the vehicle-treated endotoxin-shock-mice (**Figure 6B**). H&E staining showed that PKM2-IN significantly alleviated the LPS-induced liver and lung injury (**Figure 6C**). In addition, compared with the vehicle-treated endotoxin-shock-mice, the PKM2-IN-treated endotoxin-shock-mice markedly displayed a lower percentage of apoptotic cells as observed from TUNEL staining of the lung tissue and liver tissue (**Figure 6C**).

Considering that PKM2-IN reduced the mortality and inflammatory levels in endotoxin shock, it was hypothesized that PKM2-IN may alleviate the LPS-induced endotoxin shock by inhibiting the activation of macrophages and DCs. Intriguingly, PKM2-IN significantly reduced the expressions of CD86 and CD40 on the splenic macrophages and DCs (**Figures 6D–G**).

To observe this phenomenon more intuitively, an adoptive transfer experiment was performed. The BMDMs were pretreated with the vehicle or PKM2-IN for 2 hours and labeled by CFSE before infusing them into mice. After 12 hours, LPS was injected to establish an endotoxin shock model. As shown in **Figure S9A**, compared with the no transplant control mice, mice transferred with vehicle-treated BMDMs showed a higher mortality rate; while the mortality of the mice transferred with PKM2-IN-treated BMDMs was significantly reduced compared with the mice transferred with vehicle-treated BMDMs. Similarly, compared with the no transplant control mice, mice transferred with vehicle-treated BMDMs showed a more severe histological injury in lung and liver tissues; compared with mice transferred with vehicle-treated BMDMs, the histological injury in lung and liver tissues was reduced in the mice transferred with PKM2-IN-treated BMDMs (**Figure S9B**). TUNEL staining of the lung and liver tissues illustrated that mice transferred with PKM2-IN-treated BMDMs markedly displayed a lower percentage of the apoptotic cells compared with mice transferred with vehicle-treated BMDMs (**Figure S9C**).

Of note, the activation of CFSE-labeled BMDMs, and splenic macrophages and DCs in all groups of mice were also detected. As shown in **Figures S10A, B**, CFSE^+^ BMDMs from mice transferred with PKM2-IN-treated BMDMs showed lower expression levels of CD86 and CD40 compared with that from mice transferred with vehicle-treated BMDMs. However, there were no difference of splenic macrophages and DCs activations between mice transferred with vehicle-treated BMDMs and mice transferred with PKM2-IN-treated BMDMs (**Figures S10C–F**). These data suggest that the difference of disease condition between mice transferred with vehicle-treated BMDMs and mice transferred with PKM2-IN-treated BMDMs were, at least in part, due to the activation levels of the transferred BMDMs.

### Pharmaceutical Inhibition of PKM2 Relieves the TLR7-Ligand IMQ-Induced Lupus Model

**Figure 7:**
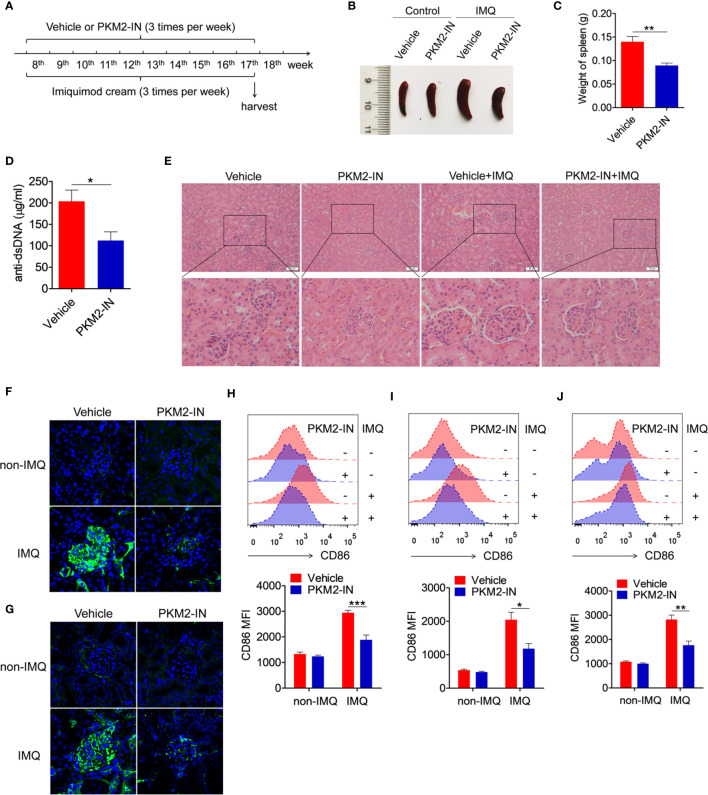
Pharmaceutical inhibition of PKM2 relieves IMQ-induced lupus model. **(A)** Treatment schematic of PKM2-IN or vehicle injection to mice following epicutaneous application of imiquimod. **(B)** Representative images of marked splenomegaly in all groups of mice. **(C)** Weight of spleens in PKM2-IN- or vehicle-treated IMQ-mice. **(D)** ELISA analysis of the anti-dsDNA antibody in serum. **(E)** H&E staining of the kidney sections from all groups of mice. **(F, G)** Immunofluorescence staining to detect IgG **(F)** and IgM **(G)** deposit in the kidney sections. **(H–J)** Flow cytometric analysis of CD86 expression on splenic macrophages **(H)**, DCs **(I)** and B cells **(J)** in all groups of mice. The data are shown as the means ± SEM (n=7 mice/group) and are representative of three independent experiments. *p < 0.05, **p < 0.01, ***p < 0.001, as determined by ANOVA tests or *t*-test.

The *in vivo* study investigated the effects of the PKM2 inhibitor on imiquimod-induced lupus-prone mice (IMQ-mice) were. Here, the skin on the right ears of the mice was treated with the TLR7-agonist imiquimod. According to the treatment schematic shown in **Figure 7A**, the therapeutic effect of PKM2-IN was designed to study the lupus symptoms in mice. After systemic treatment, under certain conditions, imiquimod induced marked splenomegaly in mice. However, the imiquimod-induced splenomegaly was significantly reversed in the PKM2-IN-treated IMQ-mice (**Figures 7B, C**). As shown in **Figure 7D**, the assessment of serum level of anti-dsDNA antibody revealed that PKM2-IN-treated IMQ-mice demonstrated a reduction in the anti-dsDNA antibody compared with that in vehicle-treated IMQ-mice. H&E staining illustrated a significant inhibition in the infiltration of the lymphoid cells as well as a diffused expansion of the mesangial matrix in the kidneys of the PKM2-IN-treated IMQ-mice when compared with that in vehicle-treated IMQ-mice (**Figure 7E**). Immunofluorescence staining of the kidney tissue revealed a relatively lesser amount of glomerular deposition of IgG (**Figure 7F**) and IgM (**Figure 7G**) in PKM2-IN-treated IMQ-mice compared with that in vehicle-treated IMQ-mice.

Further, the role of PKM2-IN in influencing the activation of macrophages, DCs and B cells was investigated in the IMQ-mice. Consistently elevated levels of the activation markers CD86 and CD40 were observed in the splenic macrophages, DCs and B cells of the vehicle-treated IMQ-mice compared with those in the untreated mice. However, PKM2-IN treatment markedly reversed IMQ-induced CD86 and CD40 expressions on the splenic macrophages (**Figures 7H** and **S11A**), DCs (**Figures 7I** and **S11B**) and B cells (**Figures 7J** and **S11C**). As is well known, abnormal germinal center plays an important role in the pathogenesis of lupus. Since PKM2 inhibitors could reduce the serum level of dsDNA in lupus mice, suggesting that PKM2-IN may affect the germinal center formation. As shown in **Figure S12**, elevated levels of the percentages of germinal center (GC) B cells (CD95^+^GL7^+^) in B220^+^ B cells and follicular helper T (Tfh) cells (CXCR5^+^PD-1^+^) in CD4^+^ T cells were observed in the spleen and mesenteric lymph nodes (mLNs) of the vehicle-treated IMQ-mice compared with those in the untreated mice. However, PKM2-IN treatment markedly reduced the percentages of GC B cells and Tfh cells in the spleens of IMQ-mice. These results indicated that blocking of the PKM2 activity relieved the symptoms of the IMQ-mice.

### Pharmaceutical Inhibition of PKM2 Relieves the Lupus-Symptoms in MRL/*lpr* Mice

**Figure 8:**
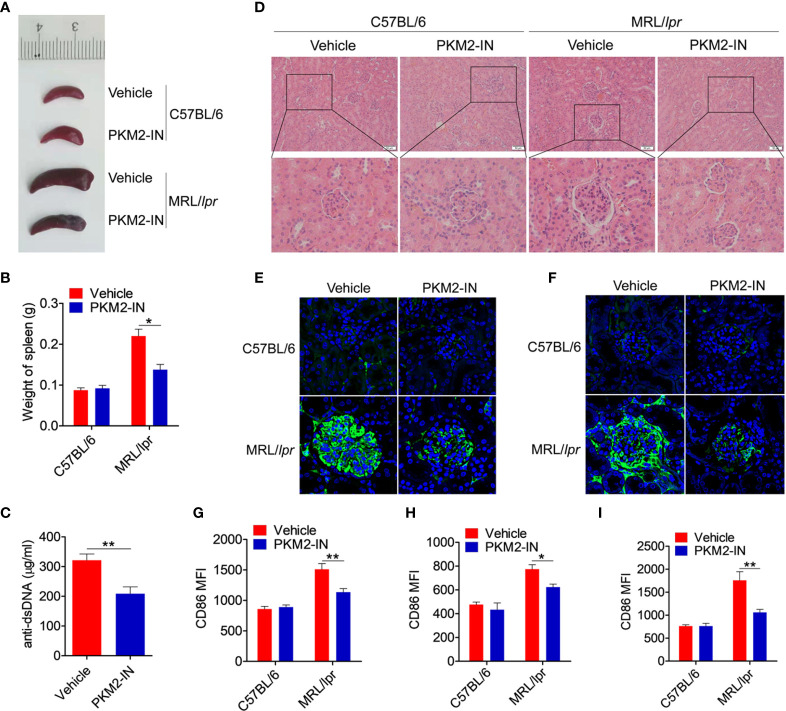
Pharmaceutical inhibition of PKM2 relieves the lupus-symptoms in MRL/*lpr* mice. **(A)** Representative images of marked splenomegaly in all groups of mice. **(B)** Weight of spleen in all groups of mice. **(C)** ELISA analysis of the anti-dsDNA antibody in serum from PKM2-IN- or vehicle-treated MRL/*lpr* mice. **(D)** H&E staining of the kidney sections from all groups of mice. **(E, F)** Immunofluorescence staining to detect IgG **(E)** and IgM **(F)** deposit in the kidney sections. **(G–I)** FACS analysis of CD86 expression on splenic macrophages **(G)**, DCs **(H)** and B cells **(I)** in all groups of mice. The data are shown as the means ± SEM (n=6 mice/group) and are representative of three independent experiments. *p < 0.05, **p < 0.01, as determined by ANOVA tests or *t*-tests.

The effect of PKM2 inhibitors on the lupus symptoms in MRL/*lpr* mice was investigated in detail. Compared with the C57BL/6 mice, the vehicle-treated MRL/*lpr* mice displayed marked splenomegaly, while the PKM2-IN-treated MRL/*lpr* mice significantly reversed the results (**Figures 8A, B**). As shown in **Figure 8C**, the serum level of the anti-dsDNA antibody in the PKM2-IN-treated MRL/*lpr* mice was lower than that in the vehicle-treated MRL/*lpr* mice. Moreover, there was a significant inhibition in the infiltration of the lymphoid cells as well as a diffuse expansion of the mesangial matrix in the kidneys of the PKM2-IN-treated MRL/*lpr* mice by H&E staining when compared with that in the vehicle-treated MRL/*lpr* mice (**Figure 8D**). Immunofluorescence staining of the kidney tissue revealed a reduced amount of glomerular deposition of IgG (**Figure 8E**) and IgM (**Figure 8F**) in the PKM2-IN-treated MRL/*lpr* mice compared with that in the vehicle-treated MRL/*lpr* mice.

The effect of PKM2-IN on the activation of macrophages, DCs and B cells was analyzed in the MRL/*lpr* mice. Strikingly, the expressions of CD86 and CD40 in the splenic macrophages, DCs and B cells of vehicle-treated MRL/*lpr* mice were all significantly higher than that in the untreated mice. Moreover, PKM2-IN treatment significantly reversed the abnormal expressions of CD86 and CD40 in the macrophages (**Figures 8G** and **S13A**), DCs (**Figures 8H** and **S13B**) and B cells (**Figures 8I** and **S13C**) of MRL/*lpr* mice. In addition, PKM2-IN-treated MRL/*lpr* mice showed lower percentages of GL7^+^ GC B cells (**Figures S14A–D**) and Tfh cells (**Figures S14E–H**) in the spleen and mLNs compared with that from vehicle-treated MRL/*lpr* mice, suggesting that treatment of PKM2-IN significantly reduced the proportions of GC B cells and Tfh cells in MRL/*lpr* mice. Thus, blocking the activity of PKM2 alleviated the condition of the MRL/*lpr* mice.

To further confirm that PKM2 blockade can reverse lupus progress in the MRL/*lpr* mice, 20-week-old MRL/*lpr* mice, which have been developed spontaneous lupus-like lesions, were used to carry out the experiments. MRL/*lpr* mice and age-matched C57BL/6 mice were received intraperitoneal injections with PKM2-IN or vehicle for two weeks. As expected, compared with the C57BL/6 mice, the vehicle-treated MRL/*lpr* mice displayed marked splenomegaly, while the PKM2-IN-treated MRL/*lpr* mice significantly reversed the results (**Figures S15A, B**). As shown in **Figure S15C**, the serum level of the anti-dsDNA antibody in the PKM2-IN-treated MRL/*lpr* mice was lower than that in the vehicle-treated MRL/*lpr* mice. Moreover, there was a significant inhibition in the infiltration of the lymphoid cells as well as a diffuse expansion of the mesangial matrix in the kidneys of the PKM2-IN-treated MRL/*lpr* mice by H&E staining when compared with that in the vehicle-treated MRL/*lpr* mice (**Figure S15D**). PKM2-IN treatment significantly reversed the abnormal expressions of CD86 on splenic macrophages (**Figure S15E**), DCs (**Figure S15F**) and B cells (**Figure S15G**) of MRL/*lpr* mice. In addition, PKM2-IN-treated MRL/*lpr* mice showed lower percentages of GL7^+^ GC B cells and Tfh cells in the spleen and mLNs compared with that from vehicle-treated MRL/*lpr* mice (**Figure S16**). Overall, these findings revealed that blocking PKM2 alleviated the lupus-symptoms in the MRL/*lpr* mice. Therefore, it is worthwhile to analyze in detail the role of PKM2 as a target in the treatment of SLE.

### PKM2 Is Over-Expressed in the SLE Patients and Correlates With the Activation of the Monocytes, DCs, and B Cells

**Figure 9:**
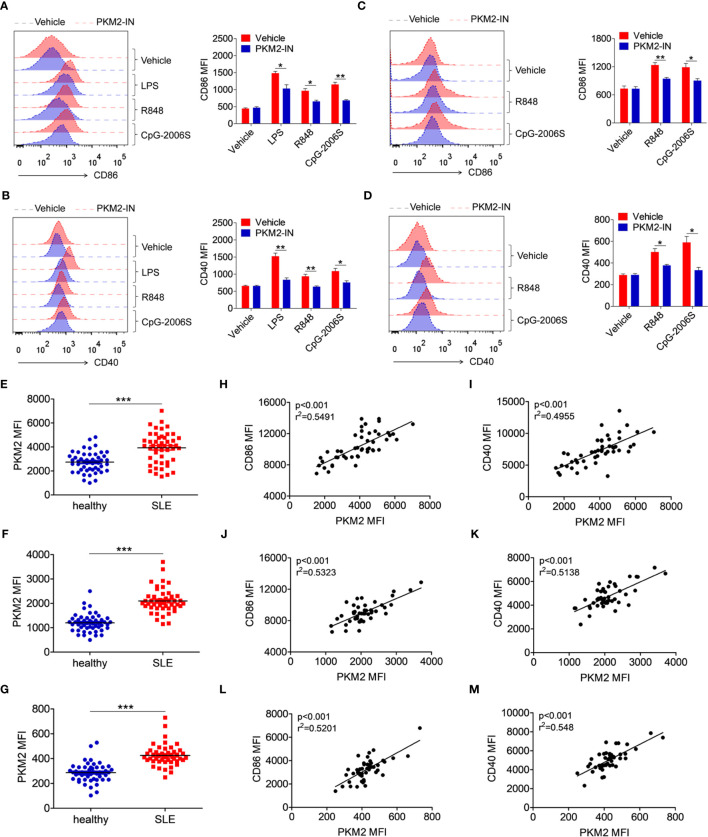
PKM2 is over-expressed in SLE patients and correlated with activation of monocytes, DCs and B cells. **(A, B)** MDMs, derived from PBMCs from healthy donors, were pretreated with PKM2-IN (5 μM) for 2 hours followed by stimulations of LPS (100 ng/mL), R848 (1 μg/mL) and CpG-2006S (1 μM). FACS analysis of CD86 and CD40 expressions on MDMs at 24 hours. **(C, D)** CD19^+^ B cells, isolated from PBMCs from healthy donors, were pretreated with PKM2-IN (5 μM) for 2 hours followed by stimulations of R848 (1 μg/mL) and CpG-2006S (1 μM). FACS analysis of CD86 and CD40 expressions on CD19^+^ B cells at 24 hours. Data are representative from one out of four biological replicates, each with three technical replicates. Error bars represent S.E.M. **(E)** FACS analysis of PKM2 protein expression on CD14^+^ monocytes in PBMCs from SLE patients (n=48) and healthy donors (n=50). **(F)** Positive correlation between PKM2 and CD86 in CD14^+^ monocytes from PBMCs of SLE patients (n=48). **(G)** Positive correlation between PKM2 and CD40 in CD14^+^ monocytes from PBMCs of SLE patients (n=48). **(H)** FACS analysis of PKM2 protein expression on CD11c^+^ dendritic cells in PBMCs from SLE patients (n=48) and healthy donors (n=50). **(I)** Positive correlation between PKM2 and CD86 in CD11c^+^ dendritic cells from PBMCs of SLE patients (n=48). **(J)** Positive correlation between PKM2 and CD40 in CD11c^+^ dendritic cells from PBMCs of SLE patients (n=48). **(K)** FACS analysis of PKM2 protein expression on CD19^+^ B cells in PBMCs from SLE patients (n=48) and healthy donors (n=50). **(L)** Positive correlation between PKM2 and CD86 in CD19^+^ B cells from PBMCs of SLE patients (n=48). **(M)** Positive correlation between PKM2 and CD40 in CD19^+^ B cells from PBMCs of SLE patients (n=48). Error bars represent S.E.M. *p < 0.05, **p < 0.01, ***p < 0.001, as determined by ANOVA tests or *t*-tests. Correlation coefficients were calculated using linear regression analysis.

To investigate whether the above phenomenon in lupus-prone mice could also extend to SLE patients, the effect of the PKM2 inhibitor PKM2-IN on the activation of TLR4, TLR7 and TLR9 pathways was investigated in the human monocytes-derived macrophages (MDMs) and CD19^+^ B cells. Consistently, PKM2-IN significantly inhibited the LPS-, R848- or CpG-2006S-induced expressions of CD86 and CD40 in human MDMs (**Figures 9A, B**) and B cells (**Figures 9C, D**).

Finally, the protein expression level of PKM2 was analyzed in the monocytes, DCs and B cells of the SLE patients. The gating strategy for CD14^+^ monocytes, CD11c^+^ dendritic cells, and CD19^+^ B cells from PBMCs of SLE patients and healthy controls was shown in **Figure S17**, as well as the representative FACS plot of PKM2. Compared with the healthy donors, the expression of PKM2 was significantly increased in the CD14^+^ monocytes (**Figure 9E**), CD11c^+^ DCs (**Figure 9F**), and CD19^+^ B cells (**Figure 9G**) of the SLE patients. Hyper-activated immune cells like the monocytes, DCs and B cells are important in the pathogenesis of SLE. The correlation between the expression levels of PKM2 and activation markers CD86 and CD40 was analyzed in the clinical samples to illuminate the relation between PKM2 and the activation of the monocytes, DCs and B cells. Interestingly, the expression levels of PKM2 significantly and positively correlated with the activation markers CD86 and CD40 in monocytes (**Figures 9H, I**), DCs (**Figures 9J, K**) and B cells (**Figures 9L, M**). These data indicated that PKM2 expression was highly elevated in the monocytes, DCs and B cells from the SLE patients compared with those from the healthy donors. Further, the PKM2 expression level positively correlated with the degree of activation of these immune cells suggesting that PKM2 played a critical role in promoting the activation of the immune cells in the SLE patients.

**Figure 10:**
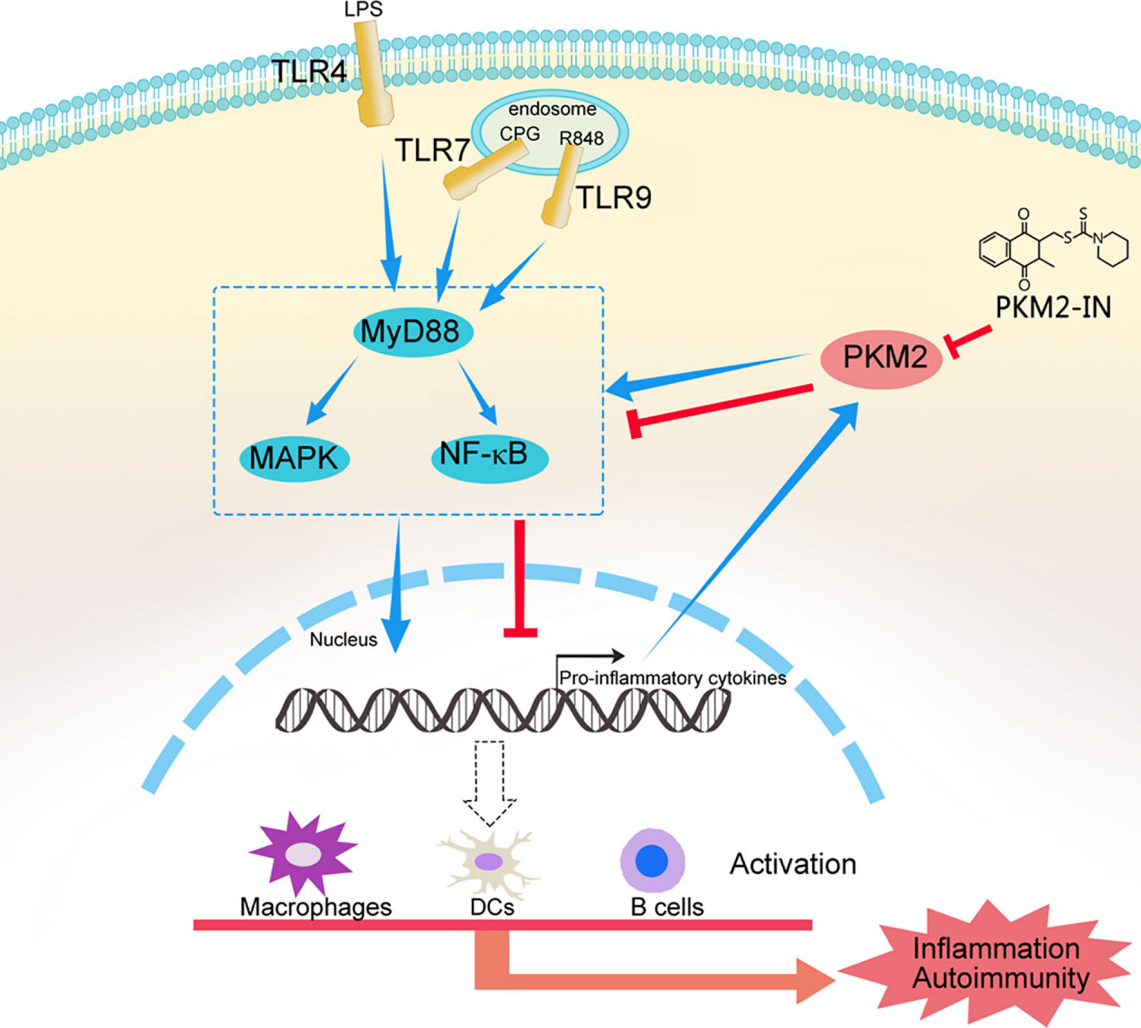
A model of the mechanism whereby PKM2 participates in the pathogenesis of TLRs-mediated inflammation and autoimmunity. This schematic shows that hyper-activation of TLRs lead to PKM2 over-expression and PKM2 augments TLR4/TLR7/TLR9-induced activations of macrophages, DCs and B cells by promoting Pyk2 activation, thereby contributing to the pathogenesis of TLRs-mediated inflammation and autoimmunity. Pharmaceutical inhibition of PKM2 by PKM2-IN can inhibit TLR4/TLR7/TLR9-induced activations of macrophages, DCs and B cells, and alleviate the pathogenesis of TLRs-mediated inflammation and autoimmunity.

Taken together, these results confirmed our hypothesis that hyper-activation of TLRs can lead to PKM2 over-expression and PKM2 augments TLR4/TLR7/TLR9-induced activations of macrophages, DCs and B cells by promoting Pyk2 activation, thereby contributing to the pathogenesis of TLRs-mediated inflammation and autoimmunity. Pharmaceutical inhibition of PKM2 by PKM2-IN can inhibit TLR4/TLR7/TLR9-induced activations of macrophages, DCs and B cells, and alleviate the pathogenesis of TLRs-mediated inflammation and autoimmunity (**Figure 10**). These findings may help us better understand the mechanism of the pathogenesis of inflammation and autoimmunity and provide a potential target to control TLR-related diseases.

## Discussion

PKM2, an important protein in the glycolytic pathway, has been shown to participate in multiple cellular processes and pathological conditions (25, 26). However, it remains unknown as to whether and how PKM2 is involved in regulating the TLR signaling pathways. In our ongoing study, we found that PKM2 contributes to the activation of the TLR signaling pathways and TLR-mediated inflammation and autoimmunity. Moreover, we also clarified the molecular mechanism of PKM2 in regulating the activation of the TLR signaling pathways.

A few studies have shown that the activation of the TLR4 signaling pathway causes the expression of PKM2 (27). Besides, PKM2 expression is widely elevated in mouse models of autoimmune diseases. Consistently, we found that the TLR4, TLR7 and TLR9 pathways induced the expression of PKM2 in macrophages, DCs and B cells under *in vivo* and *in vitro* conditions. This observation indicated that TLRs were responsible for the expression of PKM2. It is worth noting that PKM2 expression has not yet been reported in patients with inflammatory and autoimmune diseases. In this study, we analyzed the clinical samples and found that the PKM2 expression was abnormally elevated in patients with SLE, and was positively correlated with the degree of activation of the monocytes, DCs and B cells. Overall, these results indicated that the TLR-induced up-regulation of PKM2 was involved in regulating the TLR-mediated activation of the immune cells.

The *in vitro* study employed lentiviruses that up-regulate or knock down PKM2 expression to evaluate the function of PKM2 in the activation of the TLR pathways. The results indicated that the over-expression of PKM2 promoted the activation of the TLR pathways, while interference with PKM2 expression inhibited the activation of the TLR pathways. In contrast, in the *in vivo* studies, the PKM2 inhibitor PKM2-IN was employed to explore the effects of PKM2 in the mice models of TLR-mediated endotoxin shock and lupus. We believe that it will be more meaningful to use PKM2 gene-deficient mice to study the impact of PKM2 on the pathogenesis of inflammation and autoimmunity.

Previous studies suggest that Pyk2 plays a vital role in TLR activation (23, 24). We found that PKM2 significantly regulated the TLR-mediated phosphorylation of Pyk2. To explore the mechanism of PKM2 in the activation of Pyk2, we conducted Co-IP experiments and found no direct interaction between PKM2 and Pyk2. PKM2 is an important molecule in the glycolysis process (28, 29). Thus, inhibition of PKM2 significantly inhibited the production of ATP by glycolysis. Considering that ATP is essential for the activation of kinases, we speculated that PKM2 promotes the activation of Pyk2 by promoting glycolysis and producing ATP. This hypothesis needs more studies for verification.

Intriguingly, we found that the acute inflammation and lupus mice symptoms were substantially ameliorated by the PKM2 inhibitor PKM2-IN. Therefore, PKM2 should be explored as a potential novel therapeutic target in inflammation and autoimmune diseases. In summary, further elucidation of the PKM2 function should eventually allow their manipulation in the treatment strategies for various diseases intimately associated with the TLR signaling pathways. Further research is required to clarify these issues.

## Data Availability Statement

The raw data supporting the conclusions of this article will be made available by the authors, without undue reservation.

## Ethics Statement

The studies involving human participants were reviewed and approved by the ethics committee at Jining Medical University. The patients/participants provided their written informed consent to participate in this study. The animal study was reviewed and approved by Guiding Principles for the Care and Use of Laboratory Animals approved by the Jining Medica University Animal Care Committee.

## Author Contributions

XZ, YY, LJ, HZ, WZ, HZ, QM, DC, JZ, ZN, HS, and CW performed the experiments. CL, FY, MZ, JD, ZL, JM, MY, HW, and HC analyzed the data and generated figures. XZ, YY, HX, and GD wrote the manuscript. All authors contributed to the article and approved the submitted version.

## Funding

This work was supported by the National Natural Science Foundation of China (NO. 81901655 and 82071824) and the Supporting Fund for Teachers’ Research of Jining Medical University (JYFC2018FKJ030).

## Conflict of Interest

The authors declare that the research was conducted in the absence of any commercial or financial relationships that could be construed as a potential conflict of interest.
