# Supplementary material for: Pyruvate Kinase M2 Contributes to TLR-Mediated Inflammation and Autoimmunity by Promoting Pyk2 Activation
Source: Front Immunol. 2021 May 7;12:680068. doi: 10.3389/fimmu.2021.680068 (PMC8138060; doi:10.3389/fimmu.2021.680068)
Supplement: Supplementary file 1 [file DataSheet_1.pdf]

Supplemental Data

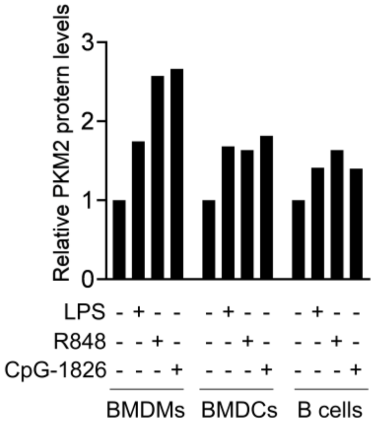

FIGURE S1 The quantification of PKM2 protein level. BMDMs, BMDCs and murine splenic B cells were stimulated with LPS (100 ng/mL), R848 (1μg/mL) and CpG-1826 (1 μM) for 24 hours and PKM2 expression was detected by western blot. Band intensities on the western blots were quantified by using ImageJ software. Data shown are representative of three independent experiments.

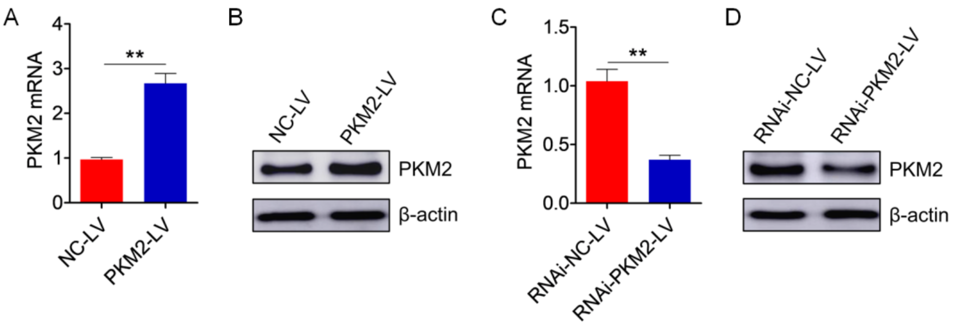

FIGURE S2 The effect of over-expression and knockdown of PKM2 in BMDMs. **(A, B)** BMDMs were infected with PKM2-LV or NC-LV for 3 days and were then harvested for qPCR and western blot analysis. qPCR analysis of PKM2 expression **(A)**. Western blot analysis of PKM2 expression **(B)**. **(C, D)** BMDMs were infected with RNAi-PKM2-LV or RNAi-NC-LV for 3 days were then harvested for qPCR and western blot analysis. qPCR analysis of PKM2 expression **(C)**. Western blot analysis of PKM2 expression **(D)**. The data shown represent the means of three independent experiments and the error bars represent the S.E.M.. **p<0.01, as determined by *t*-test.

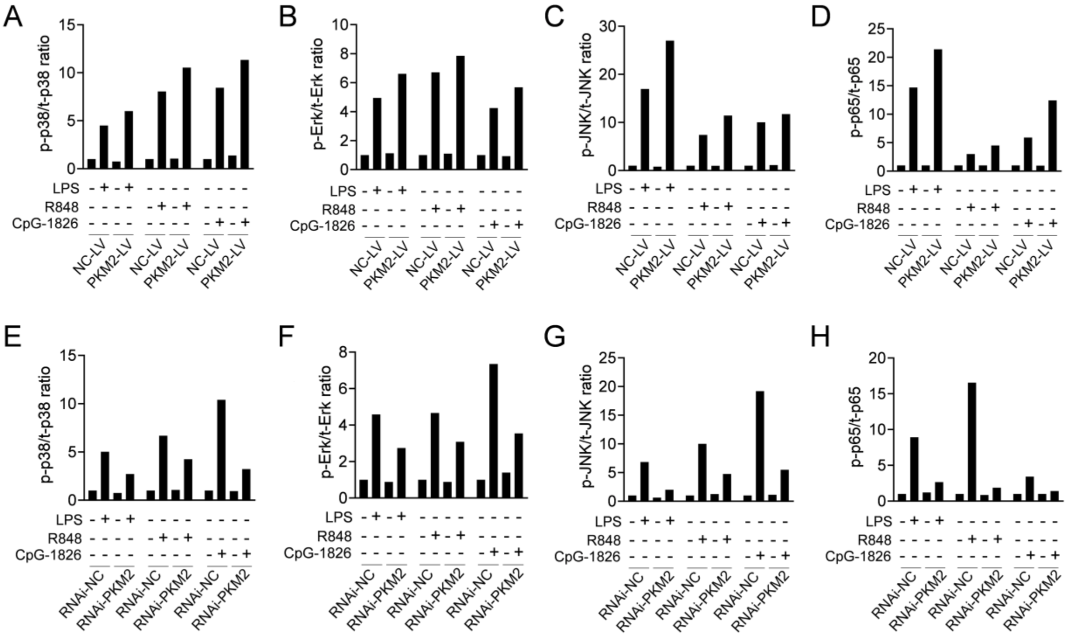

FIGURE S3 The quantification of the phosphorylation levels of p-p38, p-Erk, p-JNK and p-p65. (A-D) BMDMs, infected with lentivirus expressing PKM2 (PKM2-LV) or negative control lentivirus (NC-LV) for 3 days, were stimulated by LPS (100 ng/mL), R848 (1μg/mL) and CpG 1826 (1 μM). Western blot analysis of the phosphorylation levels of p38, Erk, JNK, and p65 at 1 hour. (E-H) BMDMs, infected with lentivirus expressing negative control-RNAi (RNAi-NC-LV) or PKM2-specific RNAi (RNAi-PKM2-LV) for 3 days, were stimulated by LPS (100 ng/mL), R848 (1μg/mL) and CpG 1826 (1 μM). Western blot analysis of the phosphorylation levels of p38, Erk, JNK, and p65 at 1 hour. Band intensities on the western blots were quantified by using ImageJ software. Data shown are representative of three independent experiments.

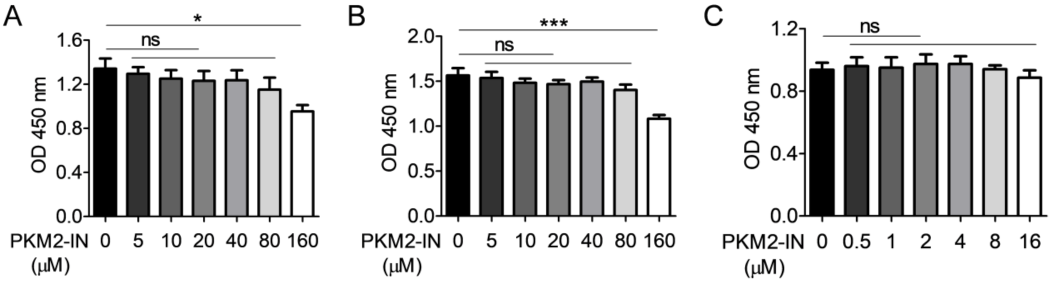

FIGURE S4 The effect of PKM2-IN on the viabilities of BMDMs, BMDCs and murine splenic B cells. BMDMs and BMDCs were treated with PKM2-IN (5, 10, 20,

40, 80 and 160 μM) and murine splenic B cells were treated with PKM2-IN (0.5, 1, 2, 4, 8 and 16 μM) for 24 hours. The viabilities of above cells were measured by using Cell counting Kit CCK-8. The data shown represent the means of three independent experiments and the error bars represent the S.E.M.. *p<0.05, ***p<0.001, as determined by ANOVA tests; ns denotes p>0.05.

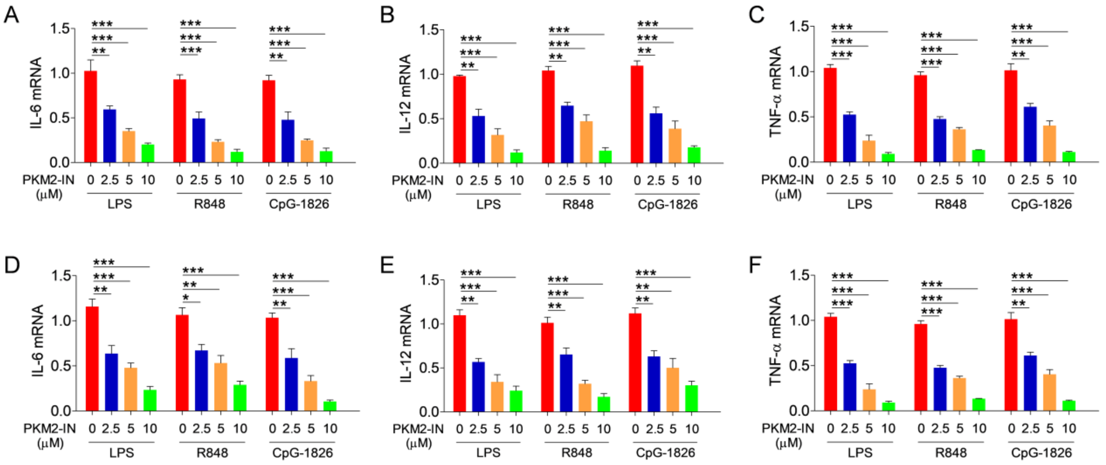

FIGURE S5 PKM2 inhibitor PKM2-IN inhibits TLR4/TLR7/TLR9-induced expressions of IL-6, IL-12 and TNF-α in BMDMs and BMDCs. (A-C) BMDMs were pretreated with PKM2-IN (2.5 μM, 5 μM and 10 μM) for 2 hours prior to stimulation of LPS (100 ng/ml), R848 (1 μg/ml), CpG-1826 (1 μM). qPCR analysis of the expressions of IL-6 (A), IL-12 (B) and TNF-α (C) at 6 hours. (D-F) BMDMs were pretreated with PKM2-IN (2.5 μM, 5 μM and 10 μM) for 2 hours prior to stimulation of LPS (100 ng/ml), R848 (1 μg/ml), CpG-1826 (1 μM). qPCR analysis of the expressions of IL-6 (D), IL-12 (E) and TNF-α (F) at 6 hours. The data shown represent the means of three independent experiments and the error bars represent the S.E.M.. *p<0.05, **p<0.001, ***p<0.01, as determined by ANOVA tests; ns denotes p>0.05.

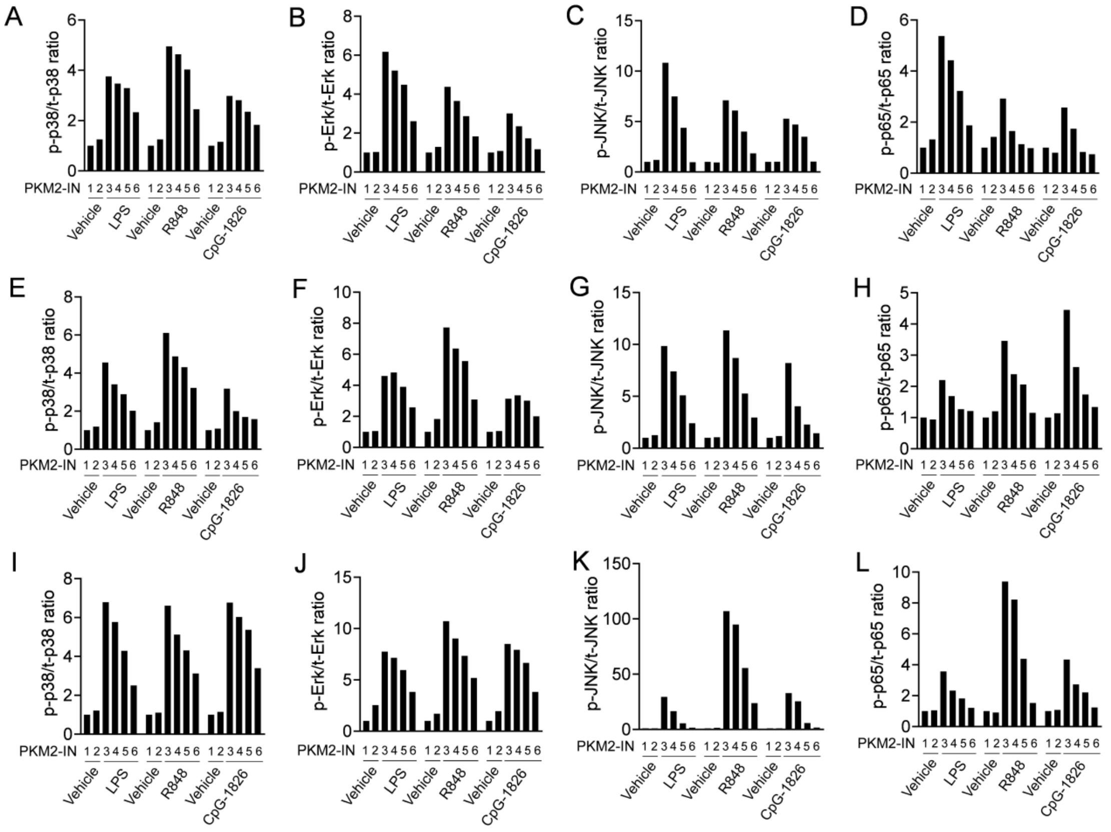

FIGURE S6 The quantification of the phosphorylation levels of p-p38, p-Erk, p-JNK and p-p65. BMDMs, BMDCs and murine splenic B cells were pretreated with PKM2 inhibitor PKM2-IN (2.5, 5 and 10 μM) for 2 hours followed by stimulations of LPS (100 ng/mL), R848 (1 μg/mL) and CpG-1826 (1 μM). Western blot analysis of the phosphorylation levels of p38, Erk, JNK, and p65 in BMDMs **(A-D)**, BMDCs **(E-H)** and murine splenic B cells **(I-L)** at 1 hour. Band intensities on the western blots were quantified by using ImageJ software. Panel 1-6 represent the concentrations of PKM2-IN is 0, 10, 0, 2.5, 5 and 10 μM, respectively. Data shown are representative of three independent experiments.

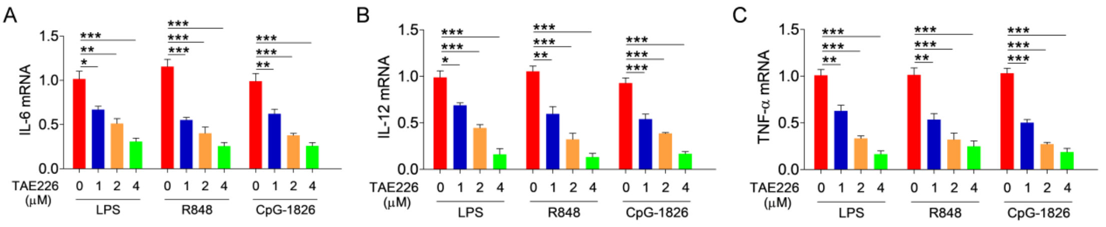

FIGURE S7 Pyk2 inhibitor TAE226 inhibits TLR4/TLR7/TLR9-induced expressions of IL-6, IL-12 and TNF-α in BMDMs. BMDMs were pretreated with

TAE226 (1 μM, 2 μM and 4 μM) for 2 hours prior to stimulation of LPS (100 ng/ml), R848 (1 μg/ml), CpG-1826 (1 μM). qPCR analysis of the expressions of IL-6 (A), IL-12 (B) and TNF-α (C) at 6 hours. The data shown represent the means of three independent experiments and the error bars represent the S.E.M.. *p<0.05, **p<0.001, ***p<0.01, as determined by ANOVA tests; ns denotes p>0.05.

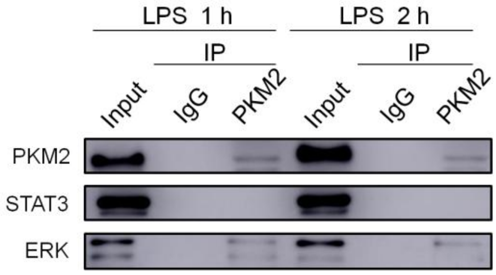

FIGURE S8 BMDMs were stimulated by LPS for 1 or 2 hours and Co-IP analysis of the interaction between PKM2 and STAT3 or Erk. Data shown are representative of three independent experiments.

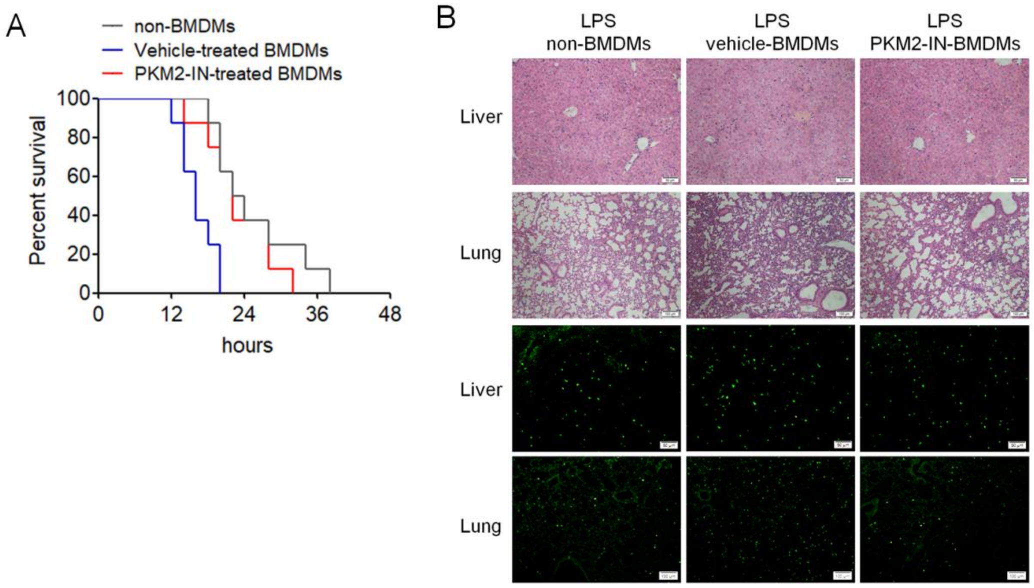

FIGURE S9 LPS-induced inflammation was reduced in mice transferred with PKM2-IN-treated BMDMs compared with vehicle-treated BMDMs. BMDMs were treated with either PKM2-IN (20 μM) or vehicle for 2 hours before being injected into C57BL/6 mice (i.p.). After 12 hours, the recipient mice were challenged by LPS (10 or 37.5 μg/g body weight). **(A)** The survivals of mice challenged by LPS (37.5 μg/g

body weight) were observed (n=8 mice/group). **(B, C)** Paraffin-embedded liver and lung sections were stained with H&E after 12 hours **(B)**. Apoptosis in the cells of liver and lung were analyzed by TUNEL after 12 hours **(C)**. The data are shown as the means ± SEM (n=6 mice/group) and are representative of three independent experiments.

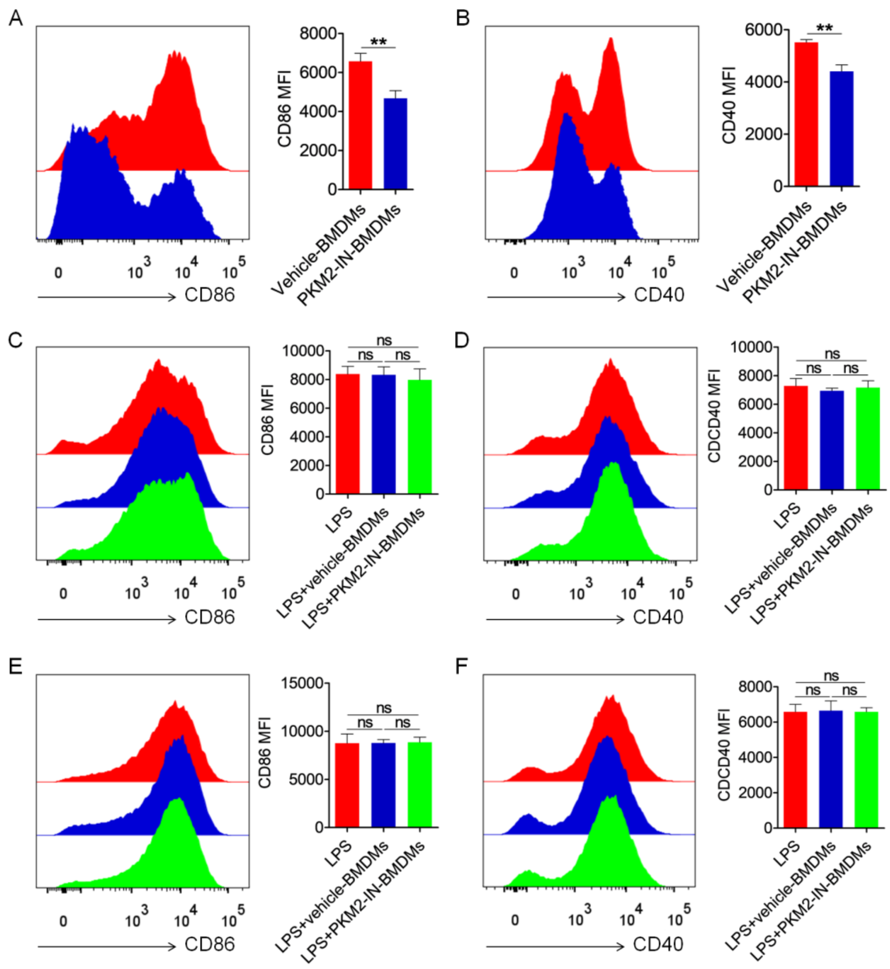

FIGURE S10 FACS analysis of the activation of macrophages and DCs in mice transferred with vehicle or PKM2-IN-treated BMDMs. **(A, B)** FACS analysis of CD86 and CD40 expressions on CFSE^{+} BMDMs isolated from the abdominal cavity of mice transferred with vehicle- or PKM2-IN-treated BMDMs. **(C, D)** FACS analysis of CD86 and CD40 expressions on splenic macrophages from all groups of mice. **(E, F)** FACS analysis of CD86 and CD40 expressions on splenic DCs from all groups of mice. The data are shown as the means ± SEM (n=6 mice/group) and are representative of three independent experiments. ***p*<0.01, as determined by ANOVA

tests or *t* test.

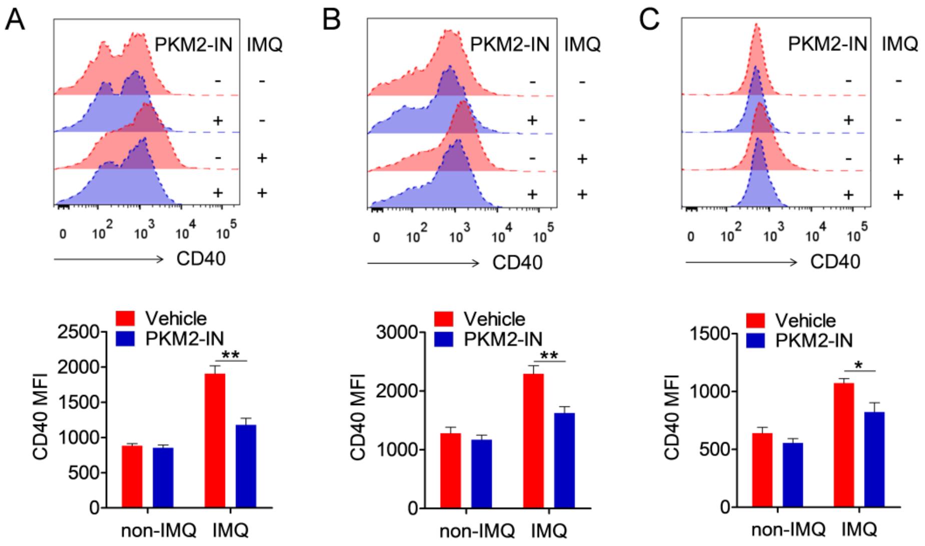

FIGURE S11 PKM2 inhibitor PKM2-IN inhibits the expression of CD40 in splenic macrophages, DCs and B cells from mice treated with IMQ. FACS analysis of CD40 expression on splenic macrophages (A), DCs (B) and B cells from all groups of mice (n=6 mice/group). The data are shown as the means ± SEM (n=6 mice/group) and are representative of three independent experiments. *p<0.05, **p<0.01, as determined by ANOVA tests.

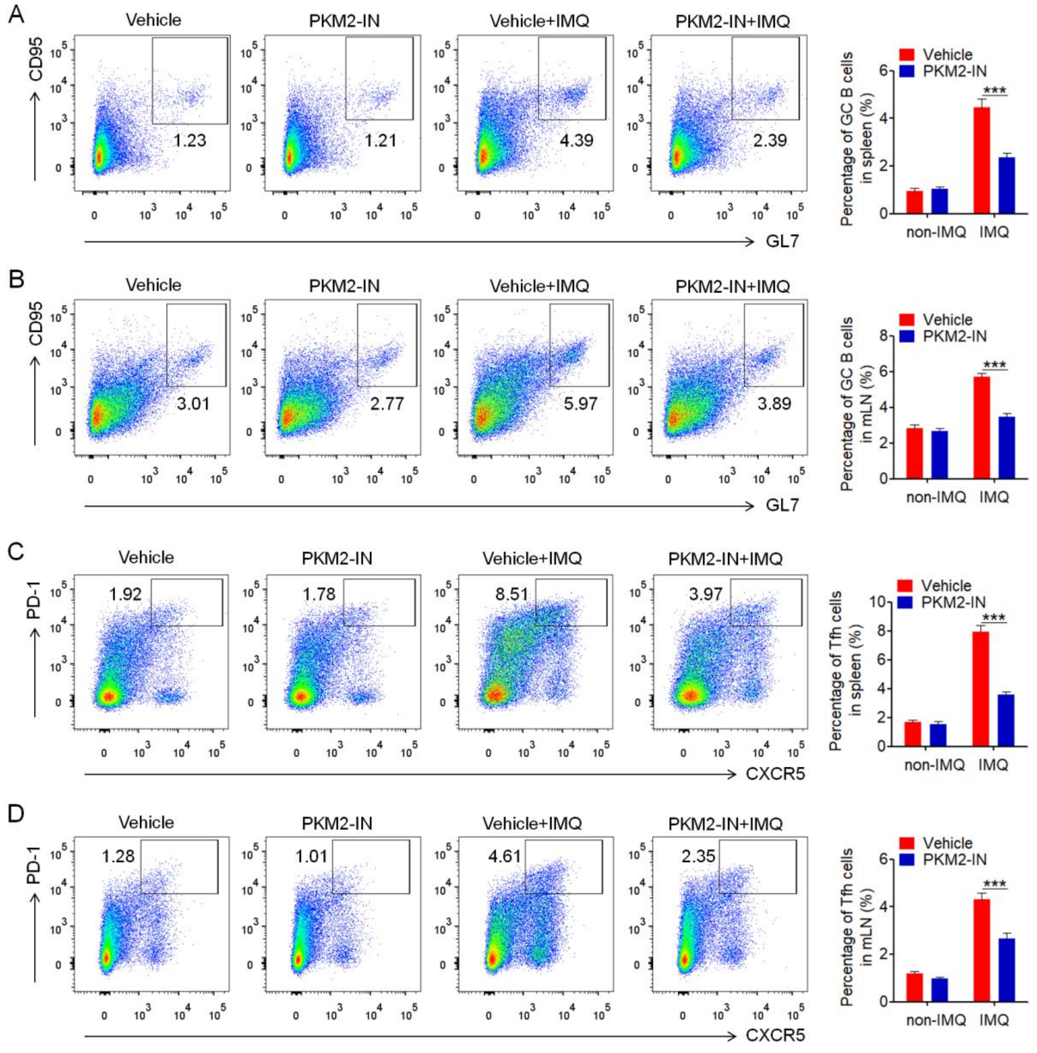

FIGURE S12 Pharmaceutical inhibition of PKM2 reverse germinal center formation in IMQ-treated mice. (**A, B**) FACS analysis of the percentages of $CD95^{+}GL7^{+}$ GC B cells in $B220^{+}$ B cells from the spleen **(A)** and mLN **(B)** of all groups of mice. (**C, D**) FACS analysis of the percentages of $CXCR5^{+}PD\text{-}1^{+}$ Tfh cells in $CD4^{+}$ B cells from the spleen **(C)** and mLN **(D)** of all groups of mice. The data are shown as the means ± SEM (n=5 mice/group) and are representative of three independent experiments. *p<0.05, as determined by ANOVA tests.

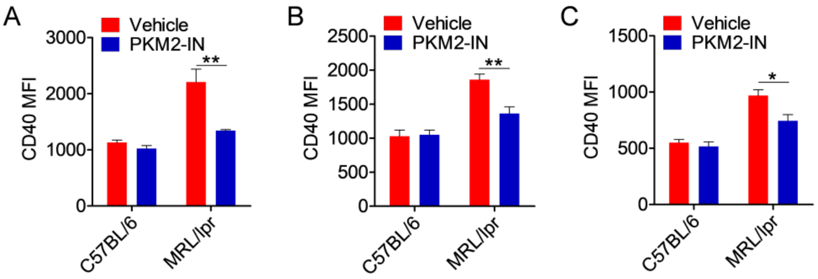

FIGURE S13 PKM2 inhibitor PKM2-IN inhibits the expression of CD40 in splenic macrophages, DCs and B cells from MRL/*lpr* mice. FACS analysis of CD40 expression on splenic macrophages (A), DCs (B) and B cells (C) from all groups of mice (n=6 mice/group). The data are shown as the means ± SEM (n=6 mice/group) and are representative of three independent experiments. *p<0.05, **p<0.01, as determined by ANOVA tests.

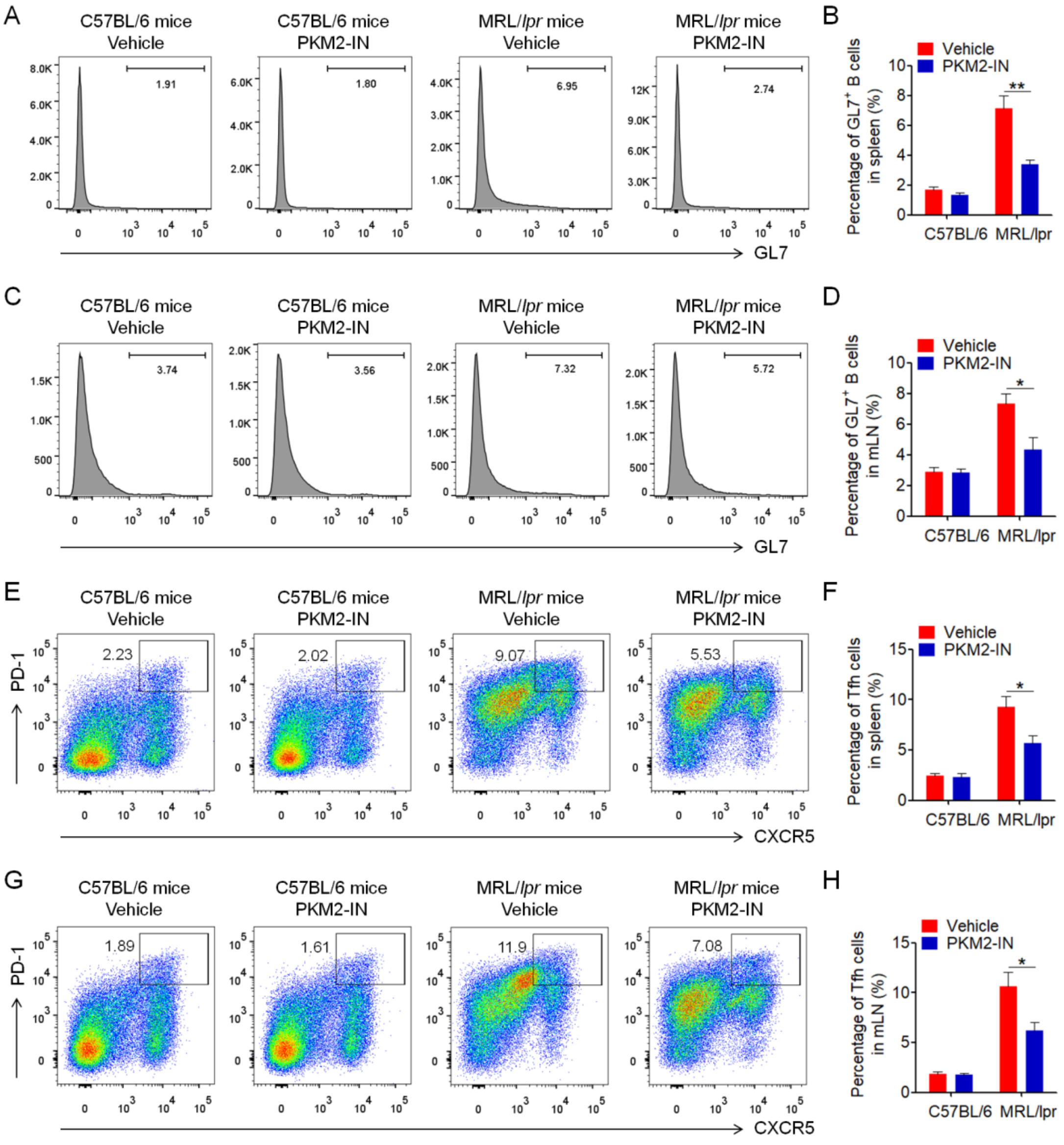

FIGURE S14 Pharmaceutical inhibition of PKM2 reduced germinal center formation in the MRL/*lpr* mice. **(A-D)** FACS analysis of the percentages of $GL7^+$ GC B cells in $B220^+$ B cells from the spleen **(A, B)** and mLN **(C, D)** of all groups of mice. **(E-H)** FACS analysis of the percentages of $CXCR5^+PD\text{-}1^+$ Tfh cells in $CD4^+$ B cells from the spleen **(E, F)** and mLN **(G, H)** of all groups of mice. The data are shown as the means ± SEM (n=5 mice/group) and are representative of three independent experiments. *p<0.05, as determined by ANOVA tests.

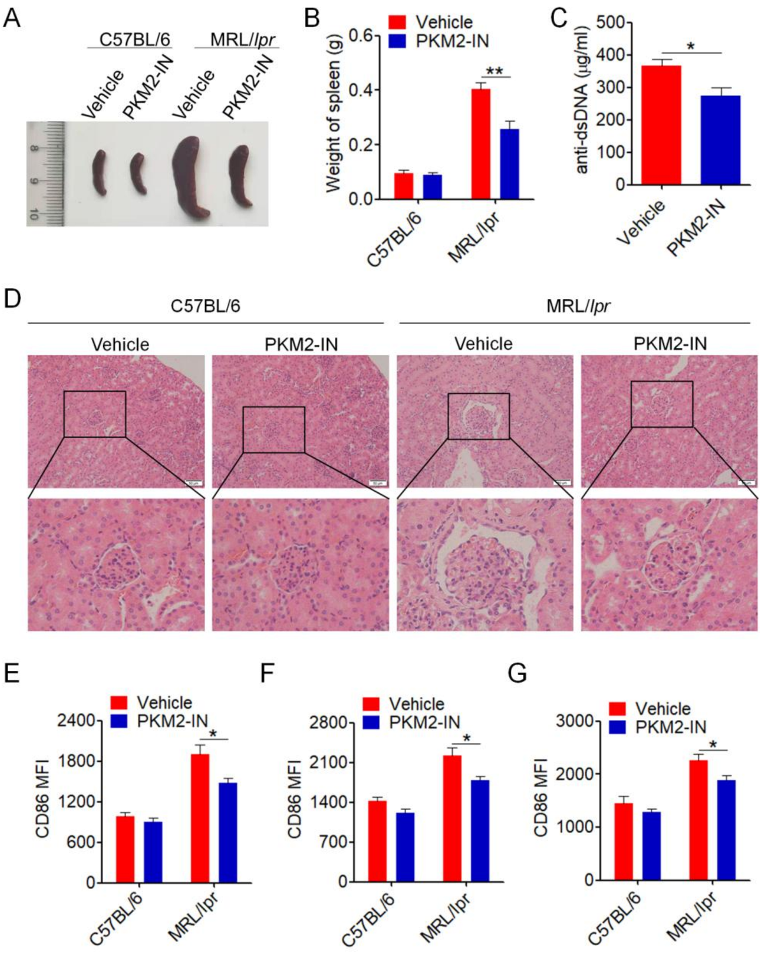

FIGURE S15 Pharmaceutical inhibition of PKM2 reverse lupus progress in the MRL/*lpr* mice. **(A)** Representative images of marked splenomegaly in all groups of mice. **(B)** Weight of spleen in all groups of mice. **(C)** ELISA analysis of the anti-dsDNA antibody in serum from PKM2-IN- or vehicle-treated MRL/*lpr* mice. **(D)** H&E staining of the kidney sections from all groups of mice. **(E-G)** FACS analysis of CD86 expression on splenic macrophages **(E)**, DCs **(F)** and B cells **(G)** in all groups of mice. The data are shown as the means ± SEM (n=5 mice/group) and are

representative of three independent experiments. *$p<0.05$, **$p<0.01$, as determined by ANOVA tests or *t*-tests.

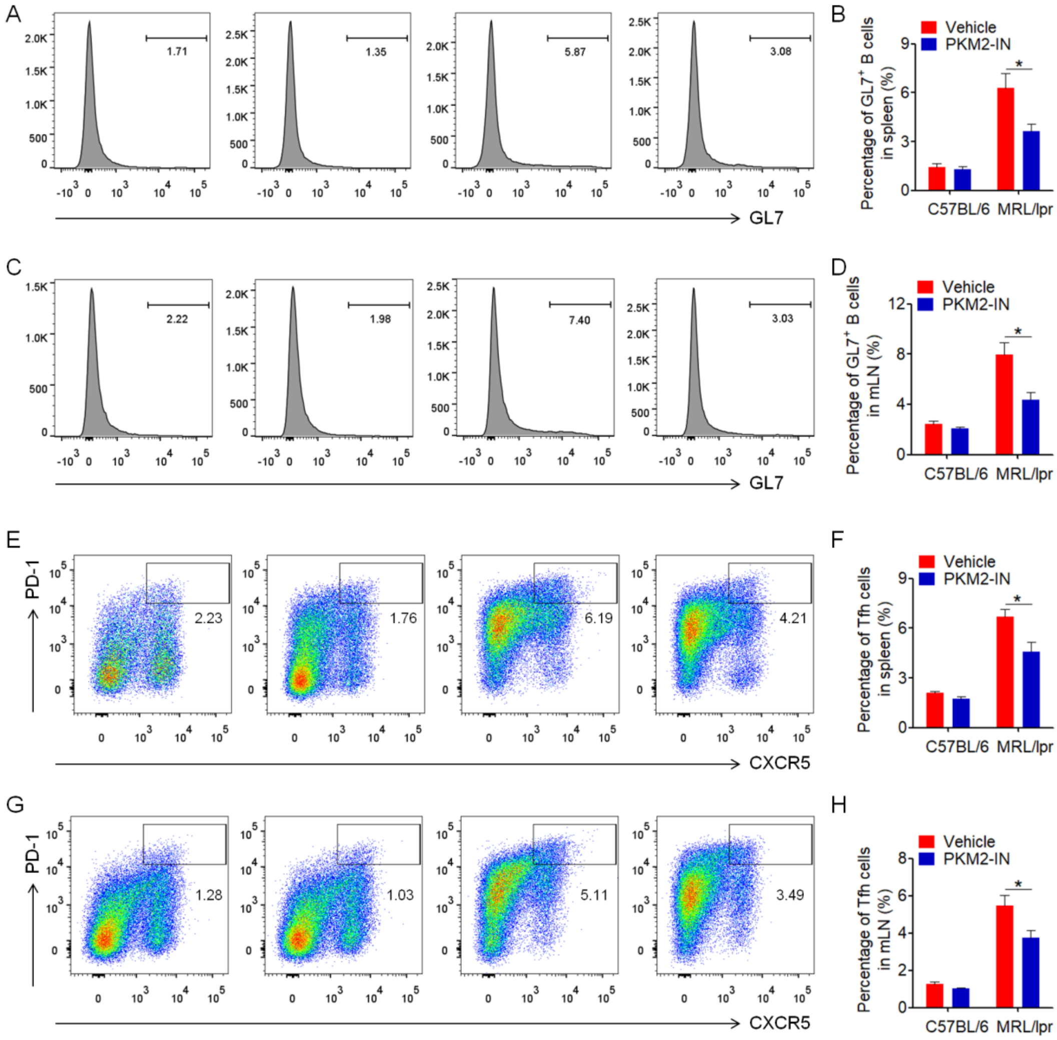

FIGURE S16 Pharmaceutical inhibition of PKM2 reverse germinal center formation in the MRL/*lpr* mice. **(A-D)** FACS analysis of the percentages of GL7$^+$ GC B cells in B220$^+$ B cells from the spleen **(A, B)** and mLN **(C, D)** of all groups of mice. **(E-H)** FACS analysis of the percentages of CXCR5$^+$PD-1$^+$ Tfh cells in CD4$^+$ B cells from the spleen **(E, F)** and mLN **(G, H)** of all groups of mice. The data are shown as the means ± SEM (n=5 mice/group) and are representative of three independent experiments. *$p<0.05$, as determined by ANOVA tests.
